# Insights into the biodegradation of two persistent fluorinated fungicides by coupling metabolic modelling with metaproteogenomics

**DOI:** 10.1038/s41598-025-31941-y

**Published:** 2026-01-07

**Authors:** Diogo A. M. Alexandrino, Miguel Semedo, Weiwei Cao, Joana Azevedo, Catarina Magalhães, Hugo Osório, Zhongjun Jia, Alexandre Campos, Ana P. Mucha, C. Marisa R. Almeida, Maria F. Carvalho

**Affiliations:** 1 Interdisciplinary Centre of Marine and Environmental Research, CIIMAR/CIMAR LA, University of Porto, Terminal de Cruzeiros do Porto de Leixões, Matosinhos, 4450- 208 Portugal; 2 ESS, Polytechnic of Porto (ESS|P.PORTO), Rua Dr. António Bernardino de Almeida 400, Porto, 4200-072 Portugal; 3 State Key Laboratory of Soil and Sustainable Agriculture, Institute of Soil Science, Chinese Academy of Sciences, Nanjing, 210008 Jiangsu Province China; 4 Faculty of Sciences, University of Porto, Rua do Campo de Alegre 790, Porto, 4150-171 Portugal; 5 i3S - Institute for Research and Innovation in Health, University of Porto, Rua Alfredo Allen 208, Porto, 4200-135 Portugal; 6 Institute of Molecular Pathology and Immunology (IPATIMUP), University of Porto, Rua Julio Amaral de Carvalho 45, Porto, 4200-135 Portugal; 7 Faculty of Medicine, University of Porto, Alameda Prof. Hernani Monteiro, Porto, 4200- 319 Portugal; 8 ICBAS - School of Medicine and Biomedical Sciences, University of Porto, Rua de Jorge Viterbo Ferreira 228, Porto, 4050-313 Portugal

**Keywords:** Epoxiconazole, Fludioxonil, Metagenomics, Metaproteomics, Defluorination, Biotechnology, Computational biology and bioinformatics, Ecology, Ecology, Environmental sciences, Microbiology

## Abstract

Epoxiconazole (EPO) and fludioxonil (FLU) are fluorinated fungicides known for their extremely high environmental persistence and significant ecotoxicological impact. Given their decades-old use in the agrochemical sector, EPO and FLU became frequent pollutants of terrestrial and aquatic ecosystems. And yet, not much is known regarding how these pesticides biodegrade in the natural environment or how to develop suitable bioremediation approaches capable of tackling their inherent recalcitrance. As such, this work focused on providing new insights into the bacterial degradation of EPO and FLU, by surveying the catabolic activity of a previously obtained EPO-enriched bacterial consortium through chemical and metaproteogenomic analyses guided by different metabolic modelling tools. The bacterial consortium was capable of extensively degrading EPO and FLU in 21 days, with fungicide removals of over 90% and defluorination efficiencies of up to 80%, but none of the subproducts predicted in silico were identified for either pesticide. Despite this, the combination of metabolic modelling tools and metaproteogenomic surveys suggested that EPO and FLU were first attacked in their N-heterocyclic moieties and that the targets of defluorination were the resulting aromatic fluorinated intermediates. This catabolic cascade is consistent with the experimental data gathered in this study and with the existing literature on this topic. Also, the degrading consortium remained stable at the taxonomical and functional levels, highlighting its catabolic plasticity in biodegrading and defluorinating two chemically distinct fluorinated compounds. This work offers a conceptual framework with novel observations that can guide future efforts to further elucidate the pathways of microbial transformation of these pesticides, ultimately contributing to better environmental risk management practices for these pollutants.

**Supplementary Information:**

The online version contains supplementary material available at 10.1038/s41598-025-31941-y.

## Introduction

Triazole and phenylpyrrole pesticides are relevant antifungal agents and established commodities in the agrochemical sector, with over 30 years of continued success in combating critical phytopathogens both in pre- and post-harvest applications^1,2^. Triazoles first emerged as safer alternatives to the first generation of azole pesticides and rapidly became established products and the dominant azole fungicides in the market^3^. Phenylpyrroles, on the other hand, are 3-cyano-4-phenylpyrrole derivatives of the pseudomonad antifungal product pyrrolnitrin, with widespread popularity as prophylactic biocides in the food and agro-industries^2^.

Epoxiconazole (EPO) and fludioxonil (FLU) stand out as key representatives of the triazole and phenylpyrrole antifungal classes, respectively, due to their high market popularity^2,4,5^. As a consequence of their prolonged market presence and global use, EPO and FLU have been detected in various environmental matrices and foodstuffs^6–9^, showing to be capable of transferring among trophic levels and of triggering significant off-target toxicity as endocrine disruptors and developmental toxicants^10–12^. In fact, the approval status of EPO has been revoked in the European Union and is being reviewed in other jurisdictions due to its potential for endocrine disruption and teratogenicity^1,13^. Aside from their confirmed role as environmental pollutants, both these fungicides showcase remarkable environmental stabilities, being recalcitrant to both physical-chemical and biological transformations^14–16^. EPO and FLU combine several structural features that largely contribute to their recalcitrance and high environmental half-lives. Unlike most triazoles, EPO harbours two haloaromatic rings and a triazole moiety, connected by an epoxide bridge, all of which are known recalcitrant functional groups^17,18^. FLU exhibits an unusual fluorination scheme (a 2,2-difluoro-1,3-benzodioxole motif) known to be resistant towards oxidative metabolism^19^, coupled to additional recalcitrant organonitrogen moieties in its molecular architecture.

As such, it comes as no surprise that only a few reports have provided robust evidence for the microbial degradation of EPO and FLU^20–22^. In addition, the underlying catabolic mechanisms remain largely unknown, despite its importance to the general understanding of how these pesticides biodegrade in the natural environment and for the development of suitable bioremediation approaches. Given this clear knowledge gap, this work focused on gaining new insights on the biodegradation of EPO and FLU by using an EPO-enriched bacterial consortium capable of biodegrading other fluoroorganics, including FLU^20,23^. For this purpose, we implemented predictive models to determine the most plausible enzymes and sub-products implicated in the microbial degradation of EPO and FLU and attempted to confirm their occurrence during the biodegradation of these fungicides by a degrading bacterial consortium, through a series of metagenomic, proteomic and chemical surveys.

## Methods

### Metabolic and enzymatic predictions

Metabolic and enzymatic predictions were performed in silico to identify possible sub-products and associated enzymes resulting from the biodegradation of EPO and FLU. To query for putative biodegradation products, BioTransformer (v3.0)^24^ and enviPath^25,26^ were used to perform metabolic predictions, using the SMILES strings of EPO or FLU as input. Predictions on BioTransformer were run using the “environmental microbial transformation” mode with a single reaction iteration, while enviPath was run under default settings. For each target fungicide, metabolic hits obtained on both tools were pooled and further enriched with additional known transformation products catalogued in the Pesticides Properties Database (PPDB)^14^. Then, compound identifiers (CID) were obtained from the PubChem database^27^ for all unique transformation products queried, for further use as input for the biocatalytic predictions using the E-Zyme2 tool from KEGG^28^. Enzymatic predictions on E-Zyme2 were conducted considering EPO or FLU as substrates for each predicted biodegradation product and all hits classified as “prokaryotic” were considered for each substrate-product pair calculated.

### Biodegradation assay

A bacterial consortium previously enriched with EPO^20^ and with capacity to biodegrade EPO, FLU and other fluorinated pesticides^23^ was used as inoculum for the biodegradation assay. Duplicate cultures of this consortium were routinely maintained in the lab in minimal salts medium (MSM) supplemented with 400 mgL^− 1^ of sodium acetate (> 99%, Sigma-Aldrich, Barcelona, Spain) once every week and 3 mgL^− 1^ of EPO (> 99%, Dr. Ehrenstofer GmbH, Augsburg, Germany) every 28 days.

For the biodegradation experiments with the target pesticides, the duplicate cultures were pooled, and 10 mL aliquots of this composite culture were used to inoculate 40 mL of sterile MSM in 100 mL borosilicate flasks. The experimental setup consisted of two sets of four-replicate experimental cultures (biodegradation cultures supplemented with EPO or FLU and control cultures with no pesticide supplementation). Biodegradation cultures were supplied once with 3 mgL^− 1^ of EPO or FLU (> 99%, Dr. Ehrenstofer GmbH, Augsburg, Germany; corresponding to 24 µL of a 5 g.L^− 1^ fungicide stock solution obtained by dissolving the proper amount of the fungicide in methanol) and weekly with sodium acetate (400 mgL^− 1^). As for control cultures, these were supplemented once with methanol (24 µL) and sodium acetate (400 mgL^− 1^) every week as well. All cultures were incubated for 28 days under static conditions, protected from light and at room temperature.

### Defluorination analyses

Defluorination was considered the primary indicator of the biodegradation of EPO and FLU. Fluoride release was determined through the potentiometric determination of the concentration of fluoride ion in cultures supernatants (3 mL) at days 0, 14 and 28, using a fluoride-selective electrode (Crison 9655 C, Crison Instruments, S.A., Barcelona, Spain). Before every potentiometric analysis, a fresh calibration curve was constructed using standard solutions of sodium fluoride (NaF) prepared in MSM, in a concentration range of 0.001–1 mM NaF. Possible interferences in the potentiometric analyses were mitigated by adjusting the ionic strength with a total ionic strength adjustment buffer (TISAB III), which was added to samples to a final concentration of 10% (v/v).

### Chemical analyses

To monitor EPO or FLU removals and attempt to detect potential biodegradation products in the culture medium, samples (1 mL) from biodegradation and control cultures were collected at days 0, 14 and 28, centrifuged (3000 x g for 15 min) and stored at −20 °C until further processing. Samples from control cultures were also analysed to compare matrices.

Prior to the analyses, samples were diluted in deionized water (in a 1:15 ratio) and subjected to solid-phase extraction (SPE) using Oasis^®^ HLB (60 mg) extraction cartridges (Waters, Massachusetts, USA) coupled to a vacuum manifold (Visiprep™ 24 SPE Vacuum Manifold, Sigma-Aldrich, Barcelona, Spain). Cartridges were conditioned with methanol (5 mL) and deionized water (5 mL), after which the diluted samples (15 mL) were loaded and washed with 5 mL of methanol (5% v/v). Cartridges were then allowed to dry for 20 min under vacuum and eluted with 1 mL of a methanol with formic acid (5% v/v). Finally, eluted solutions were vacuum-dried in a speed-vac and resuspended in methanol containing 0.1% (v/v) formic acid before analysis. With each set of samples, EPO or FLU standard solutions (3 mgL^− 1^) were freshly prepared in MSM and submitted to the same SPE protocol as the samples, to monitor the efficiency of each SPE extraction. SPE recoveries remained constant during the screening period, with average yields of > 80% for each fungicide.

Routine analyses were performed by injecting EPO or FLU standard solutions and the SPE-treated samples in a Liquid Chromatograph Thermo Finnigan Surveyor HPLC System (Thermo Scientific, MA, USA) coupled to a LCQ Fleet™ Ion Trap Mass Spectrometer (Thermo Scientific, MA, USA) (ESI-LC-MS/MS). The system was equipped with a Luna C18 column (100 mm x 4.6 mm i.d., 5 μm) from Phenomenex, which was kept at 40 °C. The mobile phase employed consisted of 25% (v/v) of methanol with 0.1% (v/v) of formic acid and the system was run isocratically. Injection volume was 10 µL at a flow rate of 0.45 mL min^− 1^. EPO and FLU were quantified by external calibration with a range of standard solutions (0.1 to 3 mgL^− 1^) prior to every analysis. The limit of detection (LOD) of the method was 6.4 µgL^− 1^ for FLU and 7.3 µgL^− 1^ for EPO.

For each fungicide, potential biodegradation products were searched following two approaches. First, an untargeted screening was conducted by looking for unique metabolic features in the biodegradation cultures (by comparison with the control cultures), as described in Alexandrino (2022)^23^. Briefly, raw files from the ESI-LC-MS/MS acquisitions (.RAW) were first converted to an appropriate file format (.mzML) using MSconvert (v.3.0.21057) and then uploaded to the XCMS platform^29^. Peak detection was performed using the matchedFilter algorithm under default parameters, followed by chromatogram alignments and the concomitant search for unique metabolic features. Metabolic features were assumed exclusive to a specific experimental condition based on a fold-change ≥ 1.5 and a p-value ≤ 0.05. Pairwise and multigroup comparisons were statistically validated through a two-tailed Student’s t-test and one-way ANOVA, respectively. A second and more targeted approach consisted on searching for suspect *m/z* signals deduced from the metabolic prediction models on the acquired ESI-LC-MS/MS data using the QualBrowser function of Xcalibur 4.1 (ThermoFisher Scientific, U.S.).

### Metagenomics analyses

Samples (5 mL) from biodegradation and control cultures were collected at days 0 and 14, centrifuged (3000 x g for 15 min) and the resulting biomass pellets were washed once in Tris-EDTA (pH 8.0) and stored at −80 °C until DNA isolation. Genomic DNA (gDNA) was extracted using the PureLink™ Microbiome DNA Purification Kit, following the instructions of the manufacturer. The extracted gDNA was then pooled according to the experimental condition, quantified in a Qubit^®^ 2.0 fluorometer (Life Technologies, CA, USA) using the Qubit^®^ dsDNA HS Assay Kit (Thermo Fisher Scientific, Waltham, MA, United States) and checked for degradation and contamination in a 1% (v/v) agarose gel. gDNA extracts suitable for shotgun metagenomic sequencing were then vacuum-dried in a speed-vac and stored at −80 °C until sequencing.

Library preparation and metagenomics sequencing were performed as described in Alexandrino (2022)^23^. Briefly, 1 µg of gDNA per sample was used for metagenomics library construction using NEBNext^®^ Ultra™ DNA Library Prep Kit for Illumina (NEB, USA). Then, each DNA sample was added index codes to attribute sequences to each sample and fragmented by sonication to a size of ~ 350 bp. The resulting fragments were end-polished, A-tailed, and ligated with the full-length adaptor for Illumina^®^ sequencing with further PCR amplification. Finally, the resulting amplified products were purified (AMPure XP system) and libraries were analysed for size distribution by Agilent2100 Bioanalyzer and quantified by real-time PCR. Paired-end sequencing was performed in an Illumina^®^ HiSeq with PE150 platform, resulting in sequencing depths of 6.04 to 8.18 Gbp (corresponding to a total data output of 57.60 Gbp). Quality control of the sequencing run was performed using Readfq V8 following default filtering parameters (https://github.com/cjfields/readfq). These sequence data have been submitted to the European Nucleotide Archive (EMBL-EBI) database under the accession numbers PRJEB56532 and PRJEB42501.

The generated metagenomes were then curated and analysed in KBase^30^. Paired-end reads were first merged and low-quality reads (< 36 bp, < Q15 within 4-base windows) were removed with Trimmomatic^31^. Read hygiene analysis was done with FastQC^32^, showing a marked improvement in read quality of the trimmed paired-end libraries. Before assembly, high-quality metagenomic reads were taxonomically assigned with the Kaiju taxonomic classifier v1.9.0^33^, using the RefSeq Genome database as reference. Database searches were performed for all taxonomic ranks in the Maximum Exact Matches mode, with no sub-sampling and considering a low abundance filter of 0.5%. For assembly, contigs were generated from the trimmed reads with the metaSPAdes assembler (v3.15.3)^34^, which were then functionally annotated with DRAM (v0.1.2)^35^. Finally, annotated metagenomes were mined for all gene products related with the enzymes predicted by E-zyme2, to narrow down the list of catabolic genes potentially available for the biodegradation of the target fungicides.

### Metaproteomics analyses

Culture aliquots of 15 mL (corresponding to biomass fresh weights (fw) of 0.03–0.25 g) were sampled from all cultures after 14 days of incubation. Biomass pellets were harvested by centrifugation (3000 x g for 15 min at 4 °C), submitted to a flash freezing step with liquid N_2_ and immediately stored at −80 °C until further processing.

Proteins were extracted in two steps to recover both hydrophilic and hydrophobic protein fractions. Extraction was initiated by homogenizing the biomass pellets in Tris-HCl (40 mM, pH 8.0), MgCl_2_ (5 mM), dithiothreitol (1 mM) and protease inhibitors (1:100, PIs, Roche, Basel, Switzerland) by bead-beating, using 2.3 mm sterile silica/zirconium beads, in a Vortex-Genie 2 (Scientific Industries, Inc., New York, USA) for 15 min, and by sonication (3 cycles of 4 s at 23 kHz). Then, samples were submitted to a denaturation step at 95 °C for 3 min and incubated in ice under orbital agitation (200 rpm) for 30 min. The lysates (hydrophylic fraction) were clarified by centrifugation (16000 x g for 20 min at 4 °C), transferred to new sterile microtubes and stored at −80 °C until peptide digestion. For the hydrophobic fraction, the cellular debris resulting from these centrifugations were solubilized in Tris-HCl (100 mM, pH 7.6), dithiothreitol (100 mM) and protease inhibitors and allowed to incubate overnight at room temperature under constant agitation (200 rpm). After this, new lysates (hydrophobic fraction) were obtained by centrifugation (16000 x g for 20 min at 4 °C), transferred to new sterile microtubes and stored at −80 °C until peptide digestion. Protein concentration of all lysates was estimated by spectrophotometry at 280 nm using a Denovix DS-11 Spectrophotometer/Fluorometer.

Lysates from both fractions were then submitted to a Filter-Aided Sample Preparation protocol^36^ and the resulting peptides were analysed in a nanoLC-MS/MS, as described in Campos et al.^37^ and Valério et al.^38^ respectively. The resulting mass spectrometry proteomics data have been deposited to the ProteomeXchange Consortium via the PRIDE partner repository with the dataset identifier PXD067833.

The generated metaproteomic datasets were further processed on the Proteome Discoverer 2.4.0.305 software (Thermo Scientific) by querying the nanoLC-MS/MS acquisitions against a custom database curated from the functional annotations of metagenomic contigs (11,333 protein-coding sequences) and against the Swiss-Prot database^39^, considering the predominant phyla of the degrading consortium (as shown by previous 16 S rRNA metabarcoding data^23^, namely Pseudomonadota (198,718 protein sequences), Bacillota (69,039 protein sequences), Actinomycetota (24,569 sequences) and Bacteroidota (6,768 protein sequences). Software parameters were as follows: tryptic peptides were identified with the SequestHT search engine; ion mass tolerance was 10 ppm and 0.02 Da for precursor and fragment ions, respectively; maximum allowed missing cleavage sites was set to 2; the following protein modifications were assumed: cysteine carbamidomethylation (constant modification) and methionine oxidation, protein N-terminus acetylation, Met-loss and Met-loss C acetyl (variable modifications); peptide confidence was set to high. The processing node Percolator was enabled with the following settings: maximum delta Cn 0.05; decoy database search target FDR 1%, validation based on q-value. For protein label free quantitation, the Minora feature detector for precursor ions was used and performed with the following parameters: unique plus razor peptides were considered for quantification and precursor abundance was based on intensity.

Differential expression analysis was performed in R (version 3.6.1) using the DEP2 package^40^ for the enzymes that were part of the metagenomic repertoire of the degrading consortia and that were actively expressed in the analysed metaproteomes. Only proteins identified in at least three of the four experimental replicates of at least one experimental condition were considered. The metaproteomic datasets were further background corrected and normalized by variance stabilizing transformation using the *normalize_vsn* function and the remaining missing values were imputed with the MinProb imputation method using the *impute* function. Then, the curated datasets were tested for differentially enriched proteins (DEPs) against the control condition using the *test_diff* function, with only the DEPs with adjusted *p*-values were below 0.05 and log_2_ fold-change ratios higher than 1.0 being considered.

## Results

### Prediction of subproducts from the biodegradation of EPO and FLU and involved enzymes

Putative biodegradation products of EPO and FLU were predicted using the BioTransformer and EnviPath tools.

For EPO, three chemically distinct subproducts were predicted by BioTransformer (two of which with no associated CID) and an additional five metabolites were estimated by EnviPath (one of which was also catalogued on PPDB) (Table S1). Most of the predicted EPO subproducts conserved the integrity of the aromatic and triazole heterocyclic rings, with the predominant biotransformations predicted being: (1) oxygen removal in the epoxy bridge, (2) dioxygenation of the chloroaromatic ring or (3) dechlorination through reductive or hydrolytic reactions. A fourth possible biotransformation route determined was the release of the triazole moiety as different carboxylated congeners, all of which feature as putative end-products.

For FLU, two sub-products were predicted by BioTransformer and another three by EnviPath (two of which were also catalogued on PPDB) (Table S2). The predicted molecules implicated the nitrile and the pyrrole functional groups as the probable biotransformation targets, with the 2,2-difluoro-1,3-benzodioxole backbone remaining intact in all predicted iterations.

Based on these metabolic predictions, for all possible pesticide-product combinations E-zyme2 returned 116 and 332 enzymes potentially involved in the initial biotransformations for EPO and FLU, respectively (Tables S3 and S4). For EPO, most of these enzymes were associated with the removal of the triazole moiety, while for FLU they were mostly implicated in the biotransformation of the pyrrole functional group.

### Biodegradation of EPO and FLU and associated consortium dynamics

**Fig. 1:**
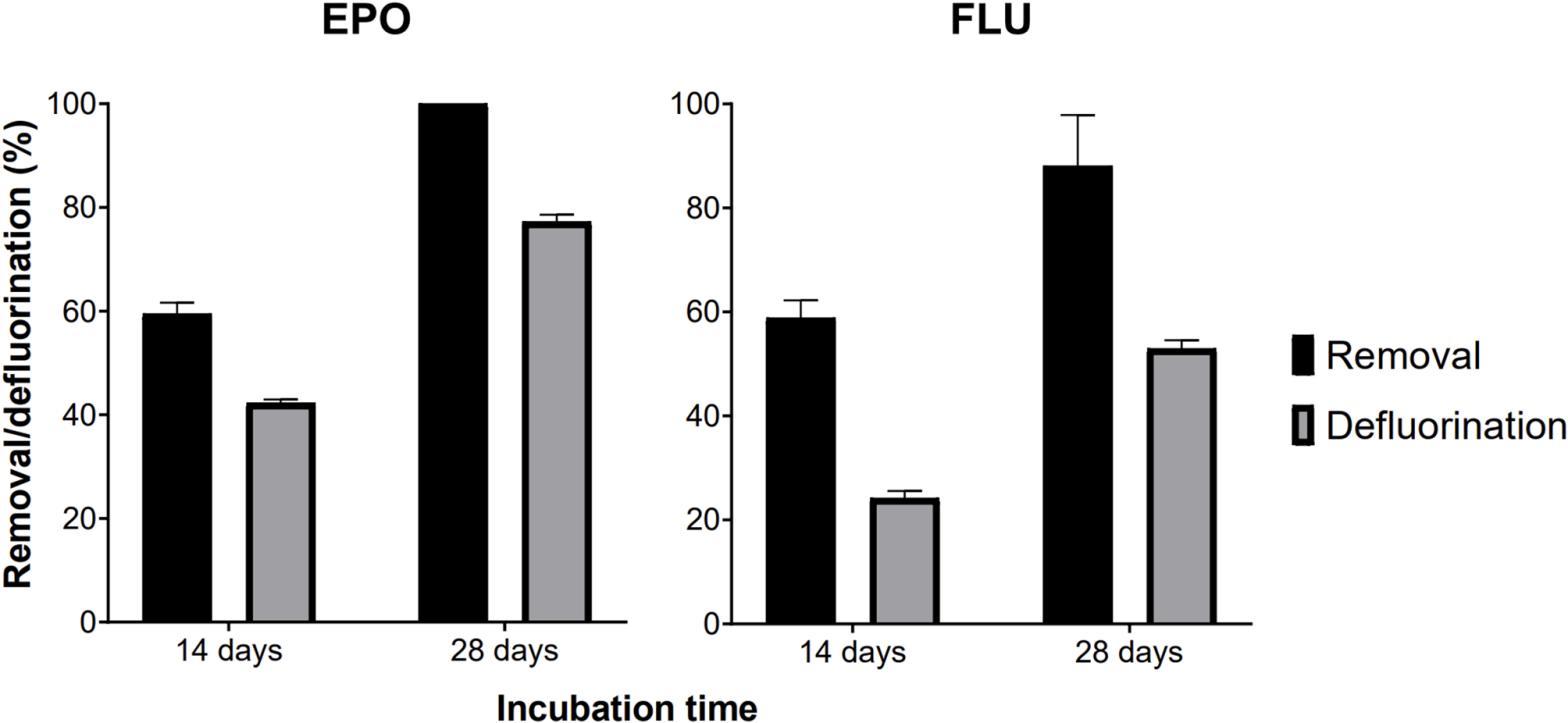
Removal and defluorination of EPO and FLU after 14 and 28 days of incubation by the degrading consortium. Bars represent mean removal/defluorination efficiencies calculated from 4 replicate cultures, while error bars show the corresponding standard deviations. Complete fungicide removal was considered when the fungicide concentration was below the LOD across all experimental replicates.

The bacterial consortium was capable of considerably biodegrading both tested fungicides, as observed by the high defluorination and compound removal efficiencies. EPO was completely removed during the 28-days incubation period, with this removal efficiency being accompanied by extensive fluoride ion release as well: 40% of the supplied fungicide was defluorinated after 14 days of incubation, an efficiency that increased almost two-fold by the end of the assay (Fig. 1). As for FLU, over 90% of this fungicide was removed from solution after 28 days, but a lower efficiency of defluorination was noted: at the 14th day of incubation, defluorination efficiencies were about 24% with an increased to ca. 55% after 28 days (Fig. 1).

Despite the recorded biodegradation performances, there were no tentative sub-products detected by ESI-LC-MS/MS. For both EPO and FLU-supplemented cultures, the predominant metabolic features differentially detected with statistical significance between the control and the biodegradation cultures corresponded to the *m/z* signals of their corresponding parental compound (*m/z* = 330.1 [M + H]^+^ for EPO and *m/z* = 247.1 [M-H]^−^ for FLU). In the EPO-degrading cultures specifically, chemical surveys also revealed an unknown product at *m/z* = 332.1 [M + H]^+^, with a similar retention time to that of EPO, that was not observed in the control cultures (Fig. S1). However, a similar MS signal was detected in the EPO standard solutions LC-MS profile as well, suggesting this was an analytical artifact correlated with the purity of the EPO reagent and not a potential degradation product. Targeting suspect *m/z* signals, derived from the metabolic predictions showed no hits as well, likely due to the low sensitivity of the ESI-LC-MS/MS equipment used in this study.

**Fig. 2:**
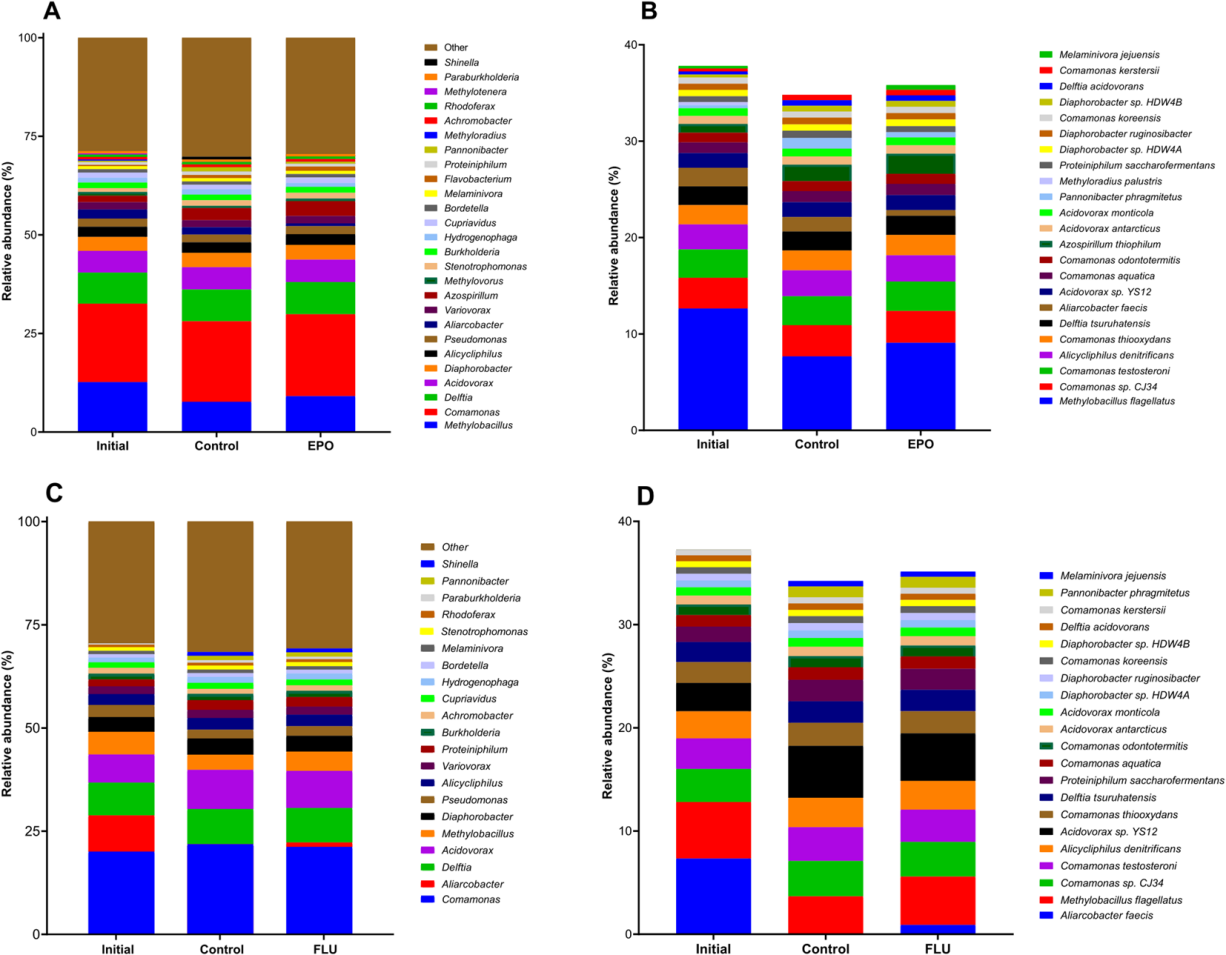
Taxonomic structure at genus (**A** and **C**) and species levels (**B** and **D**) of the degrading consortium, before and after the incubations with and without the target fungicides. Panels A-B report to the biodegradation assays with EPO while panels C-D relate to those with FLU. The “Other” category includes taxa with a relative abundance below 0.5%.

Concerning consortium dynamics, metagenome-based taxonomical assignments revealed highly structured consortia dominated by *Comamonadaceae* that remained relatively stable during the biodegradation processes (Fig. 2). In the EPO and FLU-degrading cultures, *Methylobacillus*,* Comamonas*,* Delftia* and *Acidovorax* represented over a third of the bacterial composition of the degrading consortia, though in the EPO-supplemented cultures *Comamonas* had a larger expression overall (Fig. 2A and C). At species level, *Methylobacillus flagellatus* stood out as one of the dominant species and the sole *Methylobacillus* representative in both fungicide-degrading cultures, with a higher representation in the FLU-degrading cultures (Fig. 2B and D). On the other hand, the abundance of *Comamonas*,* Delftia* and *Acidovorax* spp., the three most represented genera alongside *Methylobacillus*, was shared between different species with low individual relative abundances of (≤ 3.5%).

### Metagenomic and metaproteomic overview of predicted enzymes

**Fig. 3:**
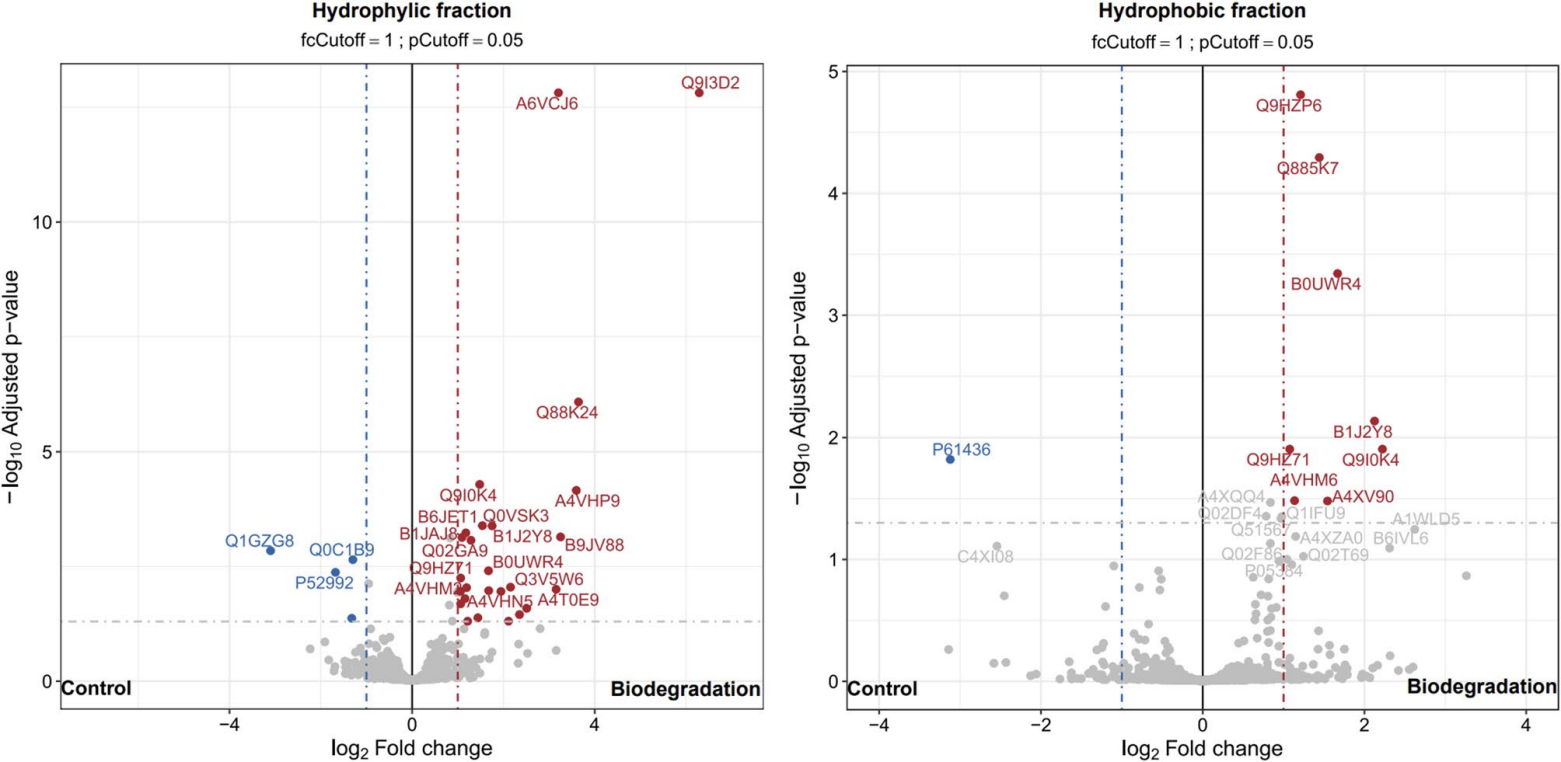
Volcano plot showing differentially expressed proteins in the metaproteomes of the degrading consortium with and without FLU supplementation.

The metagenomes of the degrading consortia obtained during the biodegradation experiment were mined using the hits provided by the E-Zyme2 predictions (Tables S3 and S4) and the occurrence of positive hits was further inspected at the metaproteome level. Of the 332 possible enzymes implicated in the biodegradation of FLU, 153 hits (corresponding to 80 unique enzymes) were detected in silico in the metagenomes (Table S5) and 34 (corresponding to 14 unique enzymes) were found to be actively expressed by the enriched consortium. However, differential expression analysis of the FLU metaproteomes did not show these predicted enzymes to be selectively enriched under FLU supplementation, in spite of the 39 proteins up-regulated under these conditions (Fig. 3).

**Fig. 4:**
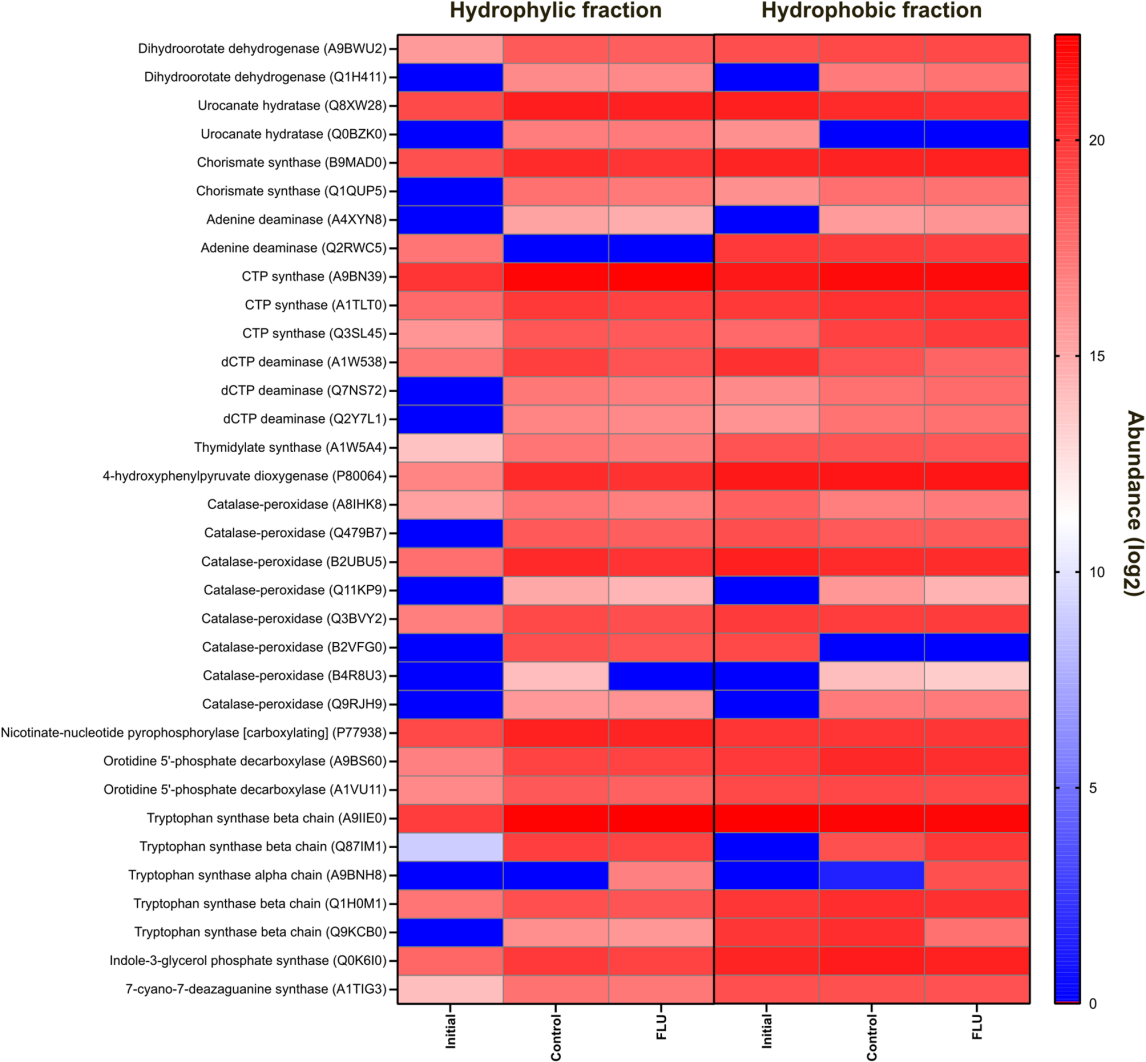
Heatmap plot comparing the log_2_-transformed abundances of predicted enzymes detected in the metaproteomes of the cultures from FLU biodegradation assay.

A finer look into the functional context of these up-regulated proteins reveals functions predominantly linked with central carbon metabolism, protein synthesis and the biosynthesis of primary metabolites. Nevertheless, a tryptophan synthase (alpha chain) enzyme (A9BNH8 by *Delftia acidovorans*), which was putatively linked to the production of two intermediates from FLU (CID2774067 or CID139594537, Table S2) was found to be exclusively expressed under FLU supplementation (Fig. 4).

**Fig. 5:**
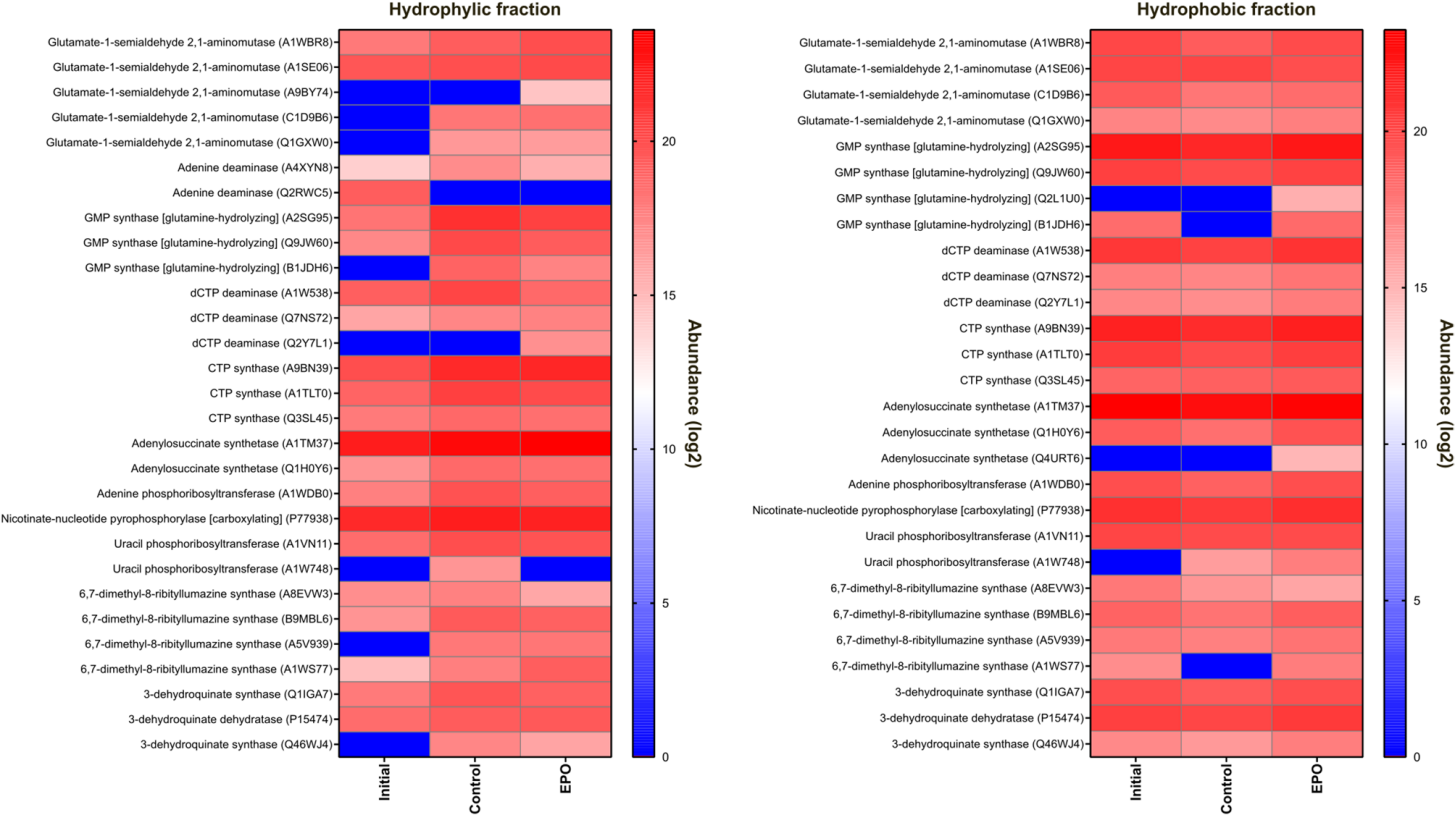
Heatmap plot comparing the log_2_-transformed abundances of predicted enzymes detected in the metaproteomes of the cultures from EPO biodegradation assay.

As for the biodegradation of EPO, of the 116 enzymes predicted using E-Zyme2, 108 (corresponding to 76 unique enzymes) were detected in silico (Table S6) and 33 (corresponding to 11 unique enzymes) were found at the metaproteome level (Fig. 5). However, there were no differentially expressed proteins detected among any of the experimental conditions related with the biodegradation of EPO (Fig. S2) and the predicted enzymes had minor shifts in expression between the control (no fungicide) and the biodegradation cultures, as well. In addition, as seen in the FLU metaproteomes, some of the predicted enzymes were exclusively detected in the presence of EPO, such as GMP synthase (Q2L1U0, expressed by *Bordetella avium*), adenylosuccinate synthetase (Q4URT6, expressed by *Xanthomonas campestris*), 6,7-dimethyl-8-ribityllumazine synthase (A1WS77, expressed by *Verminephrobacter eiseniae*) and dCTP deaminase (Q2Y7L1, expressed by *Nitrosospira multiformis*) (Fig. 5).

## Discussion

EPO and FLU are two highly relevant antifungal compounds, both in terms of their market popularity and ecotoxicity, whose biodegradation mechanisms remain to be unravelled. Despite their typically long half-lives in soils, recent reports are showing that EPO and FLU can be productively biodegraded by environmental microorganisms, leading to a significant reduction of their half-lives to non-persistent thresholds. In Alexandrino et al.^20^ we provided the first evidence of this, by showing how enriched bacterial consortia could completely degrade and defluorinate EPO and FLU in a wide range of concentrations, in some cases leading to a significant reduction of their typical half-lives. One of these bacterial consortia enriched with EPO revealed further versatility by showing the capacity to defluorinate other fluorinated pesticides, including FLU, cyhalofop-butyl and β-cyfluthrin^23^. Given its high catabolic plasticity towards fluorinated pesticides, we selected this bacterial consortium as a model system to gain more insights on the mechanisms underlying the biodegradation of EPO and FLU.

Given the scant information on the biodegradation pathways of EPO and FLU, we first resorted to well established modelling tools to highlight the most feasible biodegradation pathways for these fungicides. Based on the predicted sub-products for EPO, the metabolic models showed that arylic dechlorination or triazole displacement are the most feasible primary steps of biodegradation of this fungicide. This is congruent with the steps typically involved in the breakdown of azole fungicides, where 1,2,4-triazole arises as a frequent dead-end product^14^. As for FLU, the models suggest that the nitrile or pyrrole functional groups are the most likely targets of initial breakdown, with the 2,2-difluoro-1,3-benzodioxole backbone remaining intact. Once again, this fluorinated benzodioxole is a typical end-product of the biodegradation of other fluorinated molecules and building blocks harbouring this moiety^41^. Also, congeners of this functional group have been implicated as sub-products of the biodegradation of FLU as well^21^. Enzymatic predictions based on the estimated pesticide-product pairs further advance the possibility that N-heterocyclic moieties are the most probable sites of catabolic attack in these fungicides, given the large number of prokaryotic enzymes putatively linked to the release of the 1,2,4-triazole functional group in EPO or the degradation of the pyrrole ring in FLU.

Despite the apparent congruency of the predicted scenarios with the existing literature, we did not find experimental evidence for any of the modelled catabolic products in this study (or other potential sub-products, for that matter). The generated LC-MS datasets were screened for the typical *m/z* signals of all predicted sub-products, but no positive hits in the metabolomes of the degrading cultures were detected. There is the possibility that the implemented LC-MS strategy was not sensitive enough to detect mass peaks of metabolites that are probably being produced and released transiently and in very low concentrations to the culture medium. It is also possible that some intermediates might remain intracellularly, without being excreted into the culture medium, thus escaping detection under the applied analytical conditions. The only experimental evidence agreeing with the modelled catabolic scenarios are the fact that the defluorination of EPO and FLU was not an initial catabolic step. In fact, substantial defluorination of both fungicides during the incubation period (80% for EPO and 55% for FLU after 21 days) was detected, but their removal from solution was always faster, indicating defluorination was not the main cause of pesticide removal in this study. Nevertheless, EPO and FLU are known to resist abiotic transformations in the absence of light^14,42^ and we know from a previous study that these fungicides do not breakdown or defluorinate abiotically under the same experimental conditions used in this study^20^. This shows that the recorded degradation dynamics were bacterially mediated.

The fact that defluorination was not a primary catabolic step confirms that the targets of defluorination were fluorinated intermediates of both EPO and FLU, not the parent structures of the fungicides. Based on the metabolic predictions, these EPO fluorinated intermediates can either be dechlorinated EPO congeners or metabolites voided of the triazole moiety. Based on the experimental proteomics data obtained in this study, the most likely EPO fluorinated intermediate is one lacking the triazole functional group (e.g., 1-chloro-2-[2-(4-fluorophenyl)ethyl]benzene), as four enzymes (i.e., GMP synthase, adenylosuccinate synthetase, 6,7-dimethyl-8-ribityllumazine synthase and dCTP deaminase) linked to the release of this moiety (according to the E-Zyme2 predictions) were exclusively expressed under EPO supplementation. This is a known biotransformation route for azole fungicides, as 1,2,4-triazole has been reported as a major metabolite of over 10 different azole fungicides^14^.

For FLU, we detected a tryptophan synthase (alpha chain) exclusively expressed under FLU supplementation, which according to the metabolic predictions, can be linked to the generation of 2,2-difluoro-1,3-benzodioxole derivatives from FLU. Despite the lack of signals of any of these 2,2-difluoro-1,3-benzodioxole congeners in the LC-MS datasets, the possibility of this biotransformation route gains strength in light of the in silico predictions and on the clear pattern of differential expression of tryptophan synthases in the degrading consortium. Tryptophan synthase natively converts indole-3-glycerol to indole^43^, but the enzyme has shown broad substrate promiscuity and the ability to act on fluorinated substrates^44,45^. However, it remains to be functionally validated if the enzyme can catalyse a similar reaction focused on the pyrrole site of FLU, solely based on the pyrrole/indole high structural mimicry. Nevertheless, the feasibility of having 2,2-difluoro-1,3-benzodioxole as an intermediate in the biodegradation of FLU and as the potential defluorination target is reinforced by the fact that similar fluorinated intermediates have been implicated before in the biodegradation of this fungicide^21^ and that they can be further defluorinated into non-fluorinated products (e.g., 2,3-dihydroxybenzoic acid or pyrogallol)^21,41^. Defluorination was not a reaction predicted by the metabolic models, but for FLU the available literature suggests 2,2-difluoro-1,3-benzodioxole is defluorinated by aromatic ring hydroxylating enzymes^21,41^. Similarly for EPO, ring hydroxylation is a catabolic strategy that is probably required as well for the defluorination of the haloaromatic metabolites arising from the cleavage of the 1,2,4-triazole functional group.

During the recorded biodegradation efficiencies, the taxonomic structure of the degrading consortium remained highly stable, regardless of the presence of the fungicides and of the period of incubation, and was mostly dominated by members of the *Comamonas*, *Defltia*,* Acidovorax* and *Methylobacillus* genera. Except for *Methylobacillus*, all other mentioned bacterial genera are known to accommodate bacteria with catabolic phenotypes relevant to the biodegradation processes of EPO and FLU (e.g., dehalogenation, ring hydroxylation). Interestingly, while these genera are represented by multiple species in the consortium, *Methylobacillus flagelatus* was the sole representative of the *Methylobacillus* genus. We have shown in the past that *M. flagelatus* has a key role in the defluorination of EPO^46^ and the fact that this species had a 2-fold increase in relative abundance under EPO supplementation (when compared with FLU-supplemented cultures) seems to be suggestive of this as well. *M. flagelatus* is also the only consortium member with a relevant relative abundance that is exclusively expressing tryptophan synthase under FLU supplementation.

The lack of evident fungicide-specific shifts in consortium dynamics may be partially explained by the predominance of acetate, which represents a labile carbon source that had always been supplemented to the cultures more frequently and in higher concentrations than EPO of FLU. Still, no differences were also detected in the relative abundances of consortium members that were exclusively expressing the predicted degrading enzymes under fungicide supplementation (e.g., *D. acidovorans*,* B. avium*,* X. campestris*,* V. eiseniae*,* N. multiformis*,* A. halodurans*). The similarities in consortium composition between the EPO-supplemented and control cultures, coupled with the lack of differential expression patterns at the proteome level, indicates the consortium is highly acclimated to this fungicide. While this was expected, given the consortium was routinely cultivated with EPO in the lab prior to these experiments, this data also showed that interrupting EPO supplementation is followed by a translational lag that does not lead to rapid loss of catabolic readiness. On the other hand, the consortium structure did not differ drastically under FLU supplementation as well. While this may be masked by the frequent supplementation of acetate to these cultures, we did detect proteome shifts between FLU and EPO-supplemented cultures. As a result, this taxonomic stability of the consortium regardless of fungicide supplementation suggests that its functional architecture is similar for EPO or FLU and that it retains its key degrading taxa regardless of the fungicide being supplemented. Considering we used defluorination as the main biodegradation indicator in this study, and revisiting the presented hypothesis that defluorination of both EPO and FLU (or their corresponding fluorinated intermediates) would require bacteria with ring hydroxylating capabilities, it is possible that a core microbiome drives these relevant catabolic reactions with sufficient plasticity to act on two chemically diverse compounds. Such a degree of functional redundancy is frequently observed in single strains or mixed bacterial communities against chemically-related pollutants, but it is much less common for the biodegradation of such a chemically diverse set of compounds.

## Conclusion

This work provides a conceptual framework for the bacterial degradation of two highly persistent and commercially-relevant fluorinated fungicides, using an EPO-enriched consortium with remarkable catabolic plasticity towards fluorinated pesticides. Although no putative sub-products could be identified, the combination of metabolic modelling tools and metaproteogenomic surveys seems to suggest that the N-heterocyclic moieties of EPO and FLU are the first catabolic targets, leading to the release of aromatic fluorinated intermediates that underwent substantial defluorination by the degrading consortium. In spite of the probable chemical heterogeneity of these intermediates, the bacterial consortium was able to achieve the fungicides’ biodegradation and defluorination with minimal shifts at the taxonomic and proteomic levels. This points to high functional redundancy and catabolic plasticity of a bacterial consortium that can defuse the considerable recalcitrance typically associated with EPO and FLU. On the other hand, it also shows that the effective biodegradation of recalcitrant pollutants such as EPO and FLU may not be necessarily reliant on highly specific and (often) rare catabolic phenotypes, as it would be otherwise expected due to their xenobiotic nature. Although this study does not provide definitive evidence on the degradation pathways of EPO and FLU, it offers novel insights and critical information that can guide future efforts to elucidate the underlying mechanisms and ultimately support the development of bioremediation strategies.

## Supplementary Information

Below is the link to the electronic supplementary material.

Supplementary Material 1

## Data Availability

Sequence data have been submitted to the European Nucleotide Archive (EMBL-EBI) database under the accession numbers PRJEB56532 and PRJEB42501. The mass spectrometry metaproteomics data have been deposited to the ProteomeXchange Consortium via the PRIDE partner repository with the dataset identifier PXD067833.
