# Supplementary material for: Insights into the biodegradation of two persistent fluorinated fungicides by coupling metabolic modelling with metaproteogenomics
Source: Sci Rep. 2026 Jan 7;16:2126. doi: 10.1038/s41598-025-31941-y (PMC12808758; doi:10.1038/s41598-025-31941-y)
Supplement: Supplementary file 1 — Supplementary Material 1 [file 41598_2025_31941_MOESM1_ESM.docx]

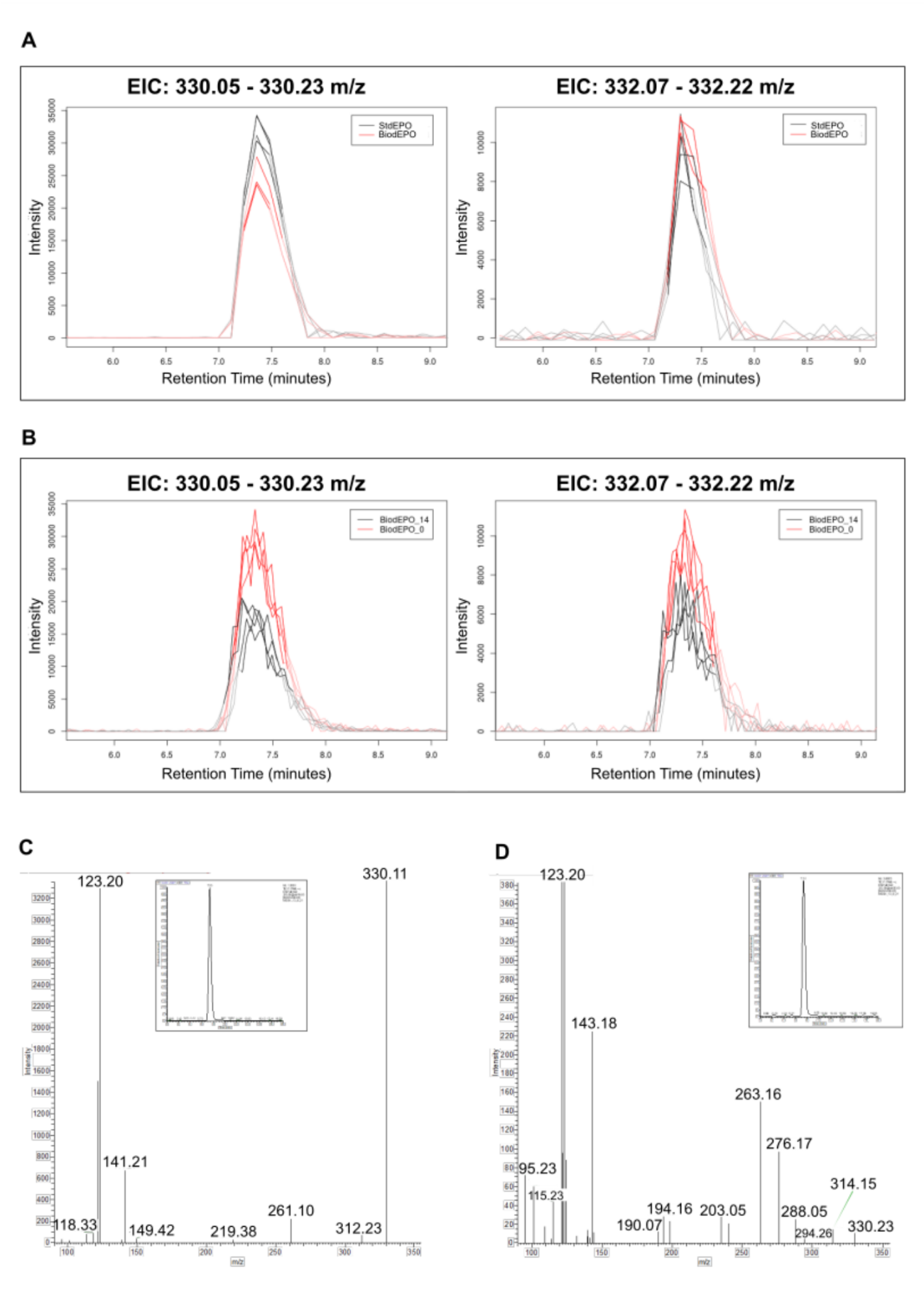


**Figure S1**. Metabolic profile of EPO biodegradation. **Panel A** shows the alignment of the ion chromatograms (EIC) from co-occurring masses (*m/z* = 330.1 [M+H]^+^ and 332.1 [M+H]^+^) from the initial samples (BiodEPO) and the 3 mgL^-1^ EPO standard solution (StdEPO); **panel B** shows the EIC alignment depicting the proportional intensity decrease of the co-occurring masses between the initial samples (BiodEPO_0) and after 14 days of incubation (BiodEPO_14) (results were not shown after 28 days of incubation, as both masses had disappeared from the solution by the end of the incubation); **panels C and D** show the fragmentation spectra of EPO and of the product with m/z = 332.1 [M+H]^+^, respectively.


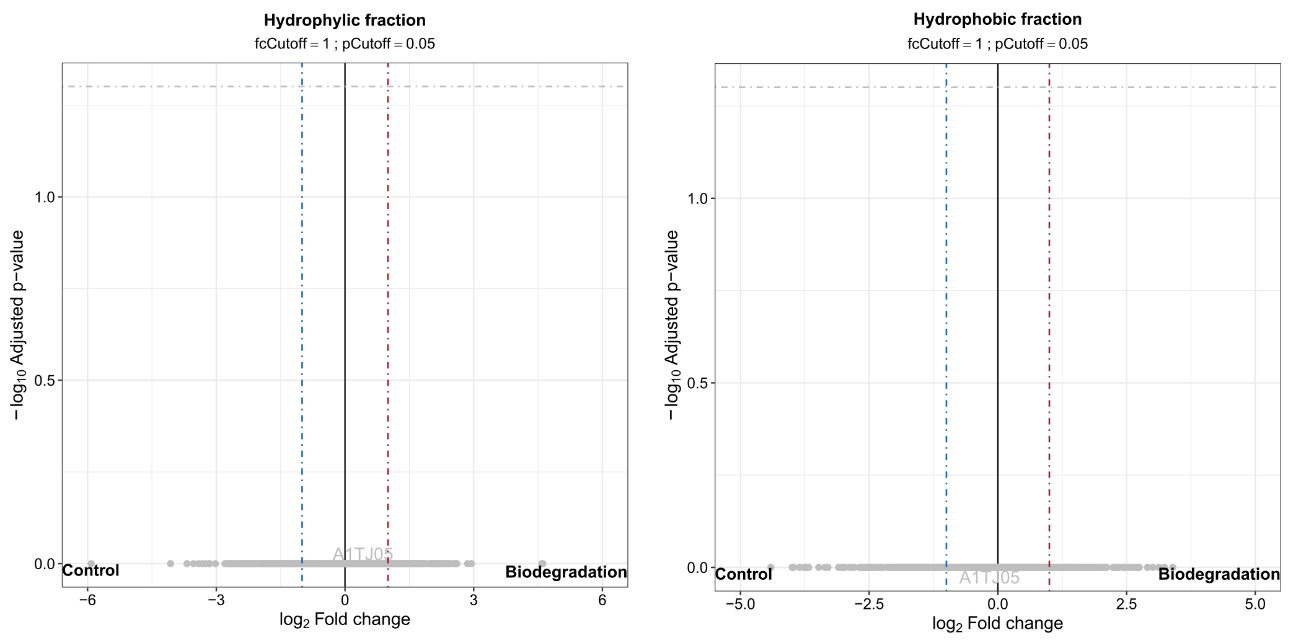


**Figure S2**. Volcano plot showing no differentially expressed proteins in the metaproteomes of the degrading consortium with and without EPO supplementation.

**Table S1.** Predicted subproducts for the biodegradation of EPO

| **Chemical structure** | **CID** | **Predictive model/Database** |
| --- | --- | --- |
|  | 10870725 | EnviPath  Pesticide Properties Database |
|  | 139597444 | EnviPath |
|  | 13602778 | BioTransformer  EnviPath |
|  | 1810180 | EnviPath |
|  | 5216290 | EnviPath |
|  | 135436542 | EnviPath |
|  | No CID^1^ | BioTransformer |
|  | No CID^1^ | BioTransformer |

^1^ Not considered for the enzymatic predictions

**Table S2.** Predicted subproducts for the biodegradation of FLU

| **Chemical structure** | **CID** | **Predictive model/Database** |
| --- | --- | --- |
|  | 139595326 | EnviPath |
|  | 139594537 | EnviPath  Pesticides Properties Database |
|  | 90135849 | BioTransformer |
|  | 153925794 | BioTransformer |
|  | 2774067 | EnviPath  Pesticides Properties Database |

**Table S3.** Output of the E-Zyme2 predictions for the biodegradation of EPO

| **KO entry** | **Gene name** | **Enzyme name [EC Number]** |
| --- | --- | --- |
| K03381 | *catA* | catechol 1,2-dioxygenase [EC:1.13.11.1] |
| K00446 | *dmpB, xylE* | catechol 2,3-dioxygenase [EC:1.13.11.2] |
| K00448 | *pcaG* | protocatechuate 3,4-dioxygenase, alpha subunit [EC:1.13.11.3] |
| K07104 | *catE* | catechol 2,3-dioxygenase [EC:1.13.11.2] |
| K00449 | *pcaH* | protocatechuate 3,4-dioxygenase, beta subunit [EC:1.13.11.3] |
| K01859 | *E5.5.1.6* | chalcone isomerase [EC:5.5.1.6] |
| K04102 | *pht5* | 4,5-dihydroxyphthalate decarboxylase [EC:4.1.1.55] |
| K04101 | *ligB* | protocatechuate 4,5-dioxygenase, beta chain [EC:1.13.11.8] |
| K15764 | *tmoE, tbuA2, touE* | toluene monooxygenase system protein E [EC:1.14.13.236 1.14.13.-] |
| K15765 | *tmoF, tbuC, touF* | toluene monooxygenase electron transfer component [EC:1.18.1.3] |
| K15766 | *absAa* | 2-aminobenzenesulfonate 2,3-dioxygenase subunit alpha [EC:1.14.12.14] |
| K01253 | *EPHX1* | microsomal epoxide hydrolase [EC:3.3.2.9] |
| K08726 | *EPHX2* | soluble epoxide hydrolase / lipid-phosphate phosphatase [EC:3.3.2.10 3.1.3.76] |
| K14579 | *nahAc, ndoB, nbzAc, dntAc* | naphthalene 1,2-dioxygenase subunit alpha [EC:1.14.12.12 1.14.12.23 1.14.12.24] |
| K14580 | *nahAd, ndoC, nbzAd, dntAd* | naphthalene 1,2-dioxygenase subunit beta [EC:1.14.12.12 1.14.12.23 1.14.12.24] |
| K14581 | *nahAa, nagAa, ndoR, nbzAa, dntAa* | naphthalene 1,2-dioxygenase ferredoxin reductase component [EC:1.18.1.7] |
| K14578 | *nahAb, nagAb, ndoA, nbzAb, dntAb* | naphthalene 1,2-dioxygenase ferredoxin component |
| K14599 | *dbfA1* | dibenzofuran dioxygenase subunit alpha [EC:1.14.12.-] |
| K14600 | *dbfA2* | dibenzofuran dioxygenase subunit beta [EC:1.14.12.-] |
| K05281 | *IFR* | 2'-hydroxyisoflavone reductase [EC:1.3.1.45] |
| K01253 | *EPHX1* | microsomal epoxide hydrolase [EC:3.3.2.9] |
| K00505 | *TYR* | tyrosinase [EC:1.14.18.1] |
| K11944 | *nidB* | PAH dioxygenase small subunit [EC:1.13.11.-] |
| K11943 | *nidA* | PAH dioxygenase large subunit [EC:1.13.11.-] |
| K12702 | *novI, CYP163A* | novobiocin biosynthesis protein NovI |
| K16421 | *hmaS, nocF* | 4-hydroxymandelate synthase [EC:1.13.11.46] |
| K04035 | *E1.14.13.81, acsF, chlE* | magnesium-protoporphyrin IX monomethyl ester (oxidative) cyclase [EC:1.14.13.81] |
| K17049 | *ebdC* | ethylbenzene hydroxylase subunit gamma |
| K17048 | *ebdB* | ethylbenzene hydroxylase subunit beta |
| K10700 | *ebdA* | ethylbenzene hydroxylase subunit alpha [EC:1.17.99.2] |
| K08689 | *bphAa, bphA1, bphA* | biphenyl 2,3-dioxygenase subunit alpha [EC:1.14.12.18] |
| K00529 | *hcaD* | 3-phenylpropionate/trans-cinnamate dioxygenase ferredoxin reductase component [EC:1.18.1.3] |
| K15750 | *bphAb, bphA2, bphE* | biphenyl 2,3-dioxygenase subunit beta [EC:1.14.12.18] |
| K14482 | *styB* | styrene monooxygenase reductase component [EC:1.5.1.-] |
| K14481 | *styA* | styrene monooxygenase [EC:1.14.14.11] |
| K10797 | *enr* | 2-enoate reductase [EC:1.3.1.31] |
| K01817 | *trpF* | phosphoribosylanthranilate isomerase [EC:5.3.1.24] |
| K13501 | *TRP1* | anthranilate synthase / indole-3-glycerol phosphate synthase / phosphoribosylanthranilate isomerase [EC:4.1.3.27 4.1.1.48 5.3.1.24] |
| K13498 | *trpCF* | indole-3-glycerol phosphate synthase / phosphoribosylanthranilate isomerase [EC:4.1.1.48 5.3.1.24] |
| K14482 | *styB* | styrene monooxygenase reductase component [EC:1.5.1.-] |
| K14481 | *styA* | styrene monooxygenase [EC:1.14.14.11] |
| K14584 | *nahD* | 2-hydroxychromene-2-carboxylate isomerase [EC:5.99.1.4] |
| K07536 | *badI* | 2-ketocyclohexanecarboxyl-CoA hydrolase [EC:3.1.2.-] |
| K07539 | *oah* | 6-oxocyclohex-1-ene-carbonyl-CoA hydrolase [EC:3.7.1.21] |
| K03336 | *iolD* | 3D-(3,5/4)-trihydroxycyclohexane-1,2-dione acylhydrolase (decyclizing) [EC:3.7.1.22] |
| K01845 | *hemL* | glutamate-1-semialdehyde 2,1-aminomutase [EC:5.4.3.8] |
| K00106 | *XDH* | xanthine dehydrogenase/oxidase [EC:1.17.1.4 1.17.3.2] |
| K13480 | *ygeU, xdhC* | xanthine dehydrogenase iron-sulfur-binding subunit |
| K11178 | *yagS* | xanthine dehydrogenase YagS FAD-binding subunit [EC:1.17.1.4] |
| K13479 | *ygeT, xdhB* | xanthine dehydrogenase FAD-binding subunit [EC:1.17.1.4] |
| K00087 | *ygeS, xdhA* | xanthine dehydrogenase molybdenum-binding subunit [EC:1.17.1.4] |
| K11177 | *yagR* | xanthine dehydrogenase YagR molybdenum-binding subunit [EC:1.17.1.4] |
| K13483 | *yagT* | xanthine dehydrogenase YagT iron-sulfur-binding subunit |
| K13482 | *xdhB* | xanthine dehydrogenase large subunit [EC:1.17.1.4] |
| K13481 | *xdhA* | xanthine dehydrogenase small subunit [EC:1.17.1.4] |
| K00546 | *HNMT* | histamine N-methyltransferase [EC:2.1.1.8] |
| K01486 | *ade* | adenine deaminase [EC:3.5.4.2] |
| K00088 | *IMPDH, guaB* | IMP dehydrogenase [EC:1.1.1.205] |
| K01489 | *cdd, CDA* | cytidine deaminase [EC:3.5.4.5] |
| K01487 | *guaD, GDA* | guanine deaminase [EC:3.5.4.3] |
| K01493 | *comEB* | dCMP deaminase [EC:3.5.4.12] |
| K01498 | *ribD1* | diaminohydroxyphosphoribosylaminopyrimidine deaminase [EC:3.5.4.26] |
| K11752 | *ribD* | diaminohydroxyphosphoribosylaminopyrimidine deaminase / 5-amino-6-(5-phosphoribosylamino)uracil reductase [EC:3.5.4.26 1.1.1.193] |
| K01488 | *add, ADA* | adenosine deaminase [EC:3.5.4.4] |
| K01951 | *guaA, GMPS* | GMP synthase (glutamine-hydrolysing) [EC:6.3.5.2] |
| K01494 | *dcd* | dCTP deaminase [EC:3.5.4.13] |
| K01937 | *pyrG, CTPS* | CTP synthase [EC:6.3.4.2] |
| K11783 | *mqnB* | futalosine hydrolase [EC:3.2.2.26] |
| K01939 | *purA, ADSS* | adenylosuccinate synthase [EC:6.3.4.4] |
| K09887 | *dcd* | dCTP deaminase (dUMP-forming) [EC:3.5.4.30] |
| K11780 | *cofG* | 7,8-didemethyl-8-hydroxy-5-deazariboflavin synthase [EC:4.3.1.32] |
| K11781 | *cofH* | 5-amino-6-(D-ribitylamino)uracil---L-tyrosine 4-hydroxyphenyl transferase [EC:2.5.1.147] |
| K11991 | *tadA* | tRNA(adenine34) deaminase [EC:3.5.4.33] |
| K00760 | *hprT, hpt, HPRT1* | hypoxanthine phosphoribosyltransferase [EC:2.4.2.8] |
| K03784 | *deoD* | purine-nucleoside phosphorylase [EC:2.4.2.1] |
| K03783 | *punA, PNP* | purine-nucleoside phosphorylase [EC:2.4.2.1] |
| K00759 | *APRT, apt* | adenine phosphoribosyltransferase [EC:2.4.2.7] |
| K16930 | *cdat8* | tRNA(cytosine8) deaminase [EC:3.5.4.35] |
| K10213 | *rihB* | ribosylpyrimidine nucleosidase [EC:3.2.2.8] |
| K00756 | *pdp* | pyrimidine-nucleoside phosphorylase [EC:2.4.2.2] |
| K00758 | *deoA, TYMP* | thymidine phosphorylase [EC:2.4.2.4] |
| K01243 | *mtnN, mtn, pfs* | adenosylhomocysteine nucleosidase [EC:3.2.2.9] |
| K00772 | *mtaP, MTAP* | 5'-methylthioadenosine phosphorylase [EC:2.4.2.28] |
| K01244 | *MTN* | 5'-methylthioadenosine nucleosidase [EC:3.2.2.16] |
| K01239 | *E3.2.2.1* | purine nucleosidase [EC:3.2.2.1] |
| K00767 | *nadC, QPRT* | nicotinate-nucleotide pyrophosphorylase (carboxylating) [EC:2.4.2.19] |
| K08728 | *E2.4.2.6* | nucleoside deoxyribosyltransferase [EC:2.4.2.6] |
| K01241 | *amn* | AMP nucleosidase [EC:3.2.2.4] |
| K15780 | *tilS-hprT* | bifunctional protein TilS/HprT [EC:6.3.4.19 2.4.2.8] |
| K00757 | *udp, UPP* | uridine phosphorylase [EC:2.4.2.3] |
| K00769 | *gpt* | xanthine phosphoribosyltransferase [EC:2.4.2.22] |
| K03816 | *xpt* | xanthine phosphoribosyltransferase [EC:2.4.2.22] |
| K03815 |  |  |
| K06164 | *phnI* | alpha-D-ribose 1-methylphosphonate 5-triphosphate synthase subunit PhnI [EC:2.7.8.37] |
| K05966 | *citG* | triphosphoribosyl-dephospho-CoA synthase [EC:2.4.2.52] |
| K06165 | *phnH* | alpha-D-ribose 1-methylphosphonate 5-triphosphate synthase subunit PhnH [EC:2.7.8.37] |
| K13927 | *citXG* | holo-ACP synthase / triphosphoribosyl-dephospho-CoA synthase [EC:2.7.7.61 2.4.2.52] |
| K06166 | *phnG* | alpha-D-ribose 1-methylphosphonate 5-triphosphate synthase subunit PhnG [EC:2.7.8.37] |
| K05780 | *phnL* | alpha-D-ribose 1-methylphosphonate 5-triphosphate synthase subunit PhnL [EC:2.7.8.37] |
| K13930 | *mdcB* | triphosphoribosyl-dephospho-CoA synthase [EC:2.4.2.52] |
| K03462 | *NAMPT* | nicotinamide phosphoribosyltransferase [EC:2.4.2.12] |
| K00768 | *E2.4.2.21, cobU, cobT* | nicotinate-nucleotide--dimethylbenzimidazole phosphoribosyltransferase [EC:2.4.2.21] |
| K04075 | *tilS, mesJ* | tRNA(Ile)-lysidine synthase [EC:6.3.4.19] |
| K13421 | *UMPS* | uridine monophosphate synthetase [EC:2.4.2.10 4.1.1.23] |
| K02825 | *pyrR* | pyrimidine operon attenuation protein / uracil phosphoribosyltransferase [EC:2.4.2.9] |
| K00761 | *upp, UPRT* | uracil phosphoribosyltransferase [EC:2.4.2.9] |
| K00763 | *pncB, NAPRT1* | nicotinate phosphoribosyltransferase [EC:6.3.4.21] |
| K00762 | *pyrE* | orotate phosphoribosyltransferase [EC:2.4.2.10] |
| K00794 | *ribH, RIB4* | 6,7-dimethyl-8-ribityllumazine synthase [EC:2.5.1.78] |
| K00793 | *ribE, RIB5* | riboflavin synthase [EC:2.5.1.9] |
| K00773 | *tgt* | queuine tRNA-ribosyltransferase [EC:2.4.2.29] |
| K00546 | *HNMT* | histamine N-methyltransferase [EC:2.1.1.8] |
| K07536 | *badI* | 2-ketocyclohexanecarboxyl-CoA hydrolase [EC:3.1.2.-] |
| K07539 | *oah* | 6-oxocyclohex-1-ene-carbonyl-CoA hydrolase [EC:3.7.1.21] |
| K03336 | *iolD* | 3D-(3,5/4)-trihydroxycyclohexane-1,2-dione acylhydrolase (decyclizing) [EC:3.7.1.22] |
| K11646 |  | 3-dehydroquinate synthase II [EC:1.4.1.24] |

**Table S4.** Output of the E-Zyme2 predictions for the biodegradation of FLU

| **KO entry** | **Gene name** | **Enzyme name [EC Number]** |
| --- | --- | --- |
| K14579 | *nahAc, ndoB, nbzAc, dntAc* | naphthalene 1,2-dioxygenase subunit alpha [EC:1.14.12.12 1.14.12.23 1.14.12.24] |
| K14580 | *nahAd, ndoC, nbzAd, dntAd* | naphthalene 1,2-dioxygenase subunit beta [EC:1.14.12.12 1.14.12.23 1.14.12.24] |
| K14581 | *nahAa, nagAa, ndoR, nbzAa, dntAa* | naphthalene 1,2-dioxygenase ferredoxin reductase component [EC:1.18.1.7] |
| K14578 | *nahAb, nagAb, ndoA, nbzAb, dntAb* | naphthalene 1,2-dioxygenase ferredoxin component |
| K08689 | *bphAa, bphA1, bphA* | biphenyl 2,3-dioxygenase subunit alpha [EC:1.14.12.18] |
| K15750 | *bphAb, bphA2, bphE* | biphenyl 2,3-dioxygenase subunit beta [EC:1.14.12.18] |
| K11944 | *nidB* | PAH dioxygenase small subunit [EC:1.13.11.-] |
| K11943 | *nidA* | PAH dioxygenase large subunit [EC:1.13.11.-] |
| K14599 | *dbfA1* | dibenzofuran dioxygenase subunit alpha [EC:1.14.12.-] |
| K14600 | *dbfA2* | dibenzofuran dioxygenase subunit beta [EC:1.14.12.-] |
| K00287 | *DHFR, folA* | dihydrofolate reductase [EC:1.5.1.3] |
| K16838 | *pucL* | urate oxidase / 2-oxo-4-hydroxy-4-carboxy-5-ureidoimidazoline decarboxylase [EC:1.7.3.3 4.1.1.97] |
| K00365 | *uaZ* | urate oxidase [EC:1.7.3.3] |
| K09461 | *E1.14.13.40* | anthraniloyl-CoA monooxygenase [EC:1.14.13.40] |
| K01712 | *hutU, UROC1* | urocanate hydratase [EC:4.2.1.49] |
| K03185 | *ubiH* | 2-octaprenyl-6-methoxyphenol hydroxylase [EC:1.14.13.-] |
| K05710 | *hcaC* | 3-phenylpropionate/trans-cinnamate dioxygenase ferredoxin component |
| K00529 | *hcaD* | 3-phenylpropionate/trans-cinnamate dioxygenase ferredoxin reductase component [EC:1.18.1.3] |
| K16016 | *rifK, asm24, asm43* | 3-amino-5-hydroxybenzoate synthase [EC:4.2.1.144 2.6.1.-] |
| K00207 | *DPYD* | dihydropyrimidine dehydrogenase (NADP+) [EC:1.3.1.2] |
| K04112 | *bcrC, badD* | benzoyl-CoA reductase subunit C [EC:1.3.7.8] |
| K04113 | *bcrB, badE* | benzoyl-CoA reductase subunit B [EC:1.3.7.8] |
| K04115 | *bcrD, badG* | benzoyl-CoA reductase subunit D [EC:1.3.7.8] |
| K04114 | *bcrA, badF* | benzoyl-CoA reductase subunit A [EC:1.3.7.8] |
| K16268 | *todC2, bedC2, tcbAb* | benzene/toluene/chlorobenzene dioxygenase subunit beta [EC:1.14.12.3 1.14.12.11 1.14.12.26] |
| K03268 | *todC1, bedC1, tcbAa* | benzene/toluene/chlorobenzene dioxygenase subunit alpha [EC:1.14.12.3 1.14.12.11 1.14.12.26] |
| K05708 | *hcaE, hcaA1* | 3-phenylpropionate/trans-cinnamate dioxygenase subunit alpha [EC:1.14.12.19] |
| K05709 | *hcaF, hcaA2* | 3-phenylpropionate/trans-cinnamate dioxygenase subunit beta [EC:1.14.12.19] |
| K14749 | *etbAb* | ethylbenzene dioxygenase subunit beta [EC:1.14.12.-] |
| K14748 | *etbAa* | ethylbenzene dioxygenase subunit alpha [EC:1.14.12.-] |
| K15250 | *BMT* | 5-hydroxyfuranocoumarin 5-O-methyltransferase [EC:2.1.1.69] |
| K15248 | *ppx-gppA* | exopolyphosphatase / guanosine-5'-triphosphate,3'-diphosphate pyrophosphatase [EC:3.6.1.11 3.6.1.40] |
| K16839 | *hpxO* | FAD-dependent urate hydroxylase [EC:1.14.13.113] |
| K05549 | *benA-xylX* | benzoate/toluate 1,2-dioxygenase subunit alpha [EC:1.14.12.10 1.14.12.-] |
| K05550 | *benB-xylY* | benzoate/toluate 1,2-dioxygenase subunit beta [EC:1.14.12.10 1.14.12.-] |
| K05784 | *benC-xylZ* | benzoate/toluate 1,2-dioxygenase reductase component [EC:1.18.1.-] |
| K13503 | *trpEG* | anthranilate synthase [EC:4.1.3.27] |
| K01657 | *trpE* | anthranilate synthase component I [EC:4.1.3.27] |
| K01658 | *trpG* | anthranilate synthase component II [EC:4.1.3.27] |
| K13501 | *TRP1* | anthranilate synthase / indole-3-glycerol phosphate synthase / phosphoribosylanthranilate isomerase [EC:4.1.3.27 4.1.1.48 5.3.1.24] |
| K13497 | *trpGD* | anthranilate synthase/phosphoribosyltransferase [EC:4.1.3.27 2.4.2.18] |
| K01656 | *TRP3* | anthranilate synthase / indole-3-glycerol phosphate synthase [EC:4.1.3.27 4.1.1.48] |
| K00254 | *DHODH, pyrD* | dihydroorotate dehydrogenase [EC:1.3.5.2] |
| K00226 | *pyrD* | dihydroorotate dehydrogenase (fumarate) [EC:1.3.98.1] |
| K02549 | *menC* | o-succinylbenzoate synthase [EC:4.2.1.113] |
| K02610 | *paaB* | ring-1,2-phenylacetyl-CoA epoxidase subunit PaaB |
| K02609 | *paaA* | ring-1,2-phenylacetyl-CoA epoxidase subunit PaaA [EC:1.14.13.149] |
| K02611 | *paaC* | ring-1,2-phenylacetyl-CoA epoxidase subunit PaaC [EC:1.14.13.149] |
| K02613 | *paaE* | ring-1,2-phenylacetyl-CoA epoxidase subunit PaaE |
| K02612 | *paaD* | ring-1,2-phenylacetyl-CoA epoxidase subunit PaaD |
| K04782 | *pchB* | isochorismate pyruvate lyase [EC:4.2.99.21] |
| K04781 | *mbtI, irp9, ybtS* | salicylate synthetase [EC:5.4.4.2 4.2.99.21] |
| K16303 | *cmtAc* | p-cumate 2,3-dioxygenase subunit beta [EC:1.14.12.25] |
| K10619 | *cmtAb* | p-cumate 2,3-dioxygenase subunit alpha [EC:1.14.12.25] |
| K02619 | *pabC* | 4-amino-4-deoxychorismate lyase [EC:4.1.3.38] |
| K03181 | *ubiC* | chorismate lyase [EC:4.1.3.40] |
| K03342 | *pabBC* | para-aminobenzoate synthetase / 4-amino-4-deoxychorismate lyase [EC:2.6.1.85 4.1.3.38] |
| K00760 | *hprT, hpt, HPRT1* | hypoxanthine phosphoribosyltransferase [EC:2.4.2.8] |
| K15936 | *urdGT2* | glycosyltransferase [EC:2.4.1.-] |
| K00088 | *IMPDH, guaB* | IMP dehydrogenase [EC:1.1.1.205] |
| K11780 | *cofG* | 7,8-didemethyl-8-hydroxy-5-deazariboflavin synthase [EC:4.3.1.32] |
| K11781 | *cofH* | 5-amino-6-(D-ribitylamino)uracil---L-tyrosine 4-hydroxyphenyl transferase [EC:2.5.1.147] |
| K03781 | *katE, CAT, catB, srpA* | catalase [EC:1.11.1.6] |
| K03782 | *katG* | catalase-peroxidase [EC:1.11.1.21] |
| K12696 | *CYP71D12* | tabersonine 16-hydroxylase [EC:1.14.14.103] |
| K13389 | *CYP80G2* | (S)-corytuberine synthase [EC:1.14.19.51] |
| K01488 | *add, ADA* | adenosine deaminase [EC:3.5.4.4] |
| K01489 | *cdd, CDA* | cytidine deaminase [EC:3.5.4.5] |
| K00767 | *nadC, QPRT* | nicotinate-nucleotide pyrophosphorylase (carboxylating) [EC:2.4.2.19] |
| K01718 | *pel* | pectate lyase [EC:4.2.2.2] |
| K16329 | *psuG* | pseudouridylate synthase [EC:4.2.1.70] |
| K01493 | *comEB* | dCMP deaminase [EC:3.5.4.12] |
| K05928 | *E2.1.1.95* | tocopherol O-methyltransferase [EC:2.1.1.95] |
| K01939 | *purA, ADSS* | adenylosuccinate synthase [EC:6.3.4.4] |
| K15933 | *lndZ5, lanZ5* | oxygenase LndZ5/LanZ5 |
| K15932 | *lndZ4, lanZ4* | reductase LndZ4/LanZ4 |
| K14257 | *ctcP, cts4, prnC* | tetracycline 7-halogenase / FADH2 O2-dependent halogenase [EC:1.14.19.49 1.14.19.-] |
| K01494 | *dcd* | dCTP deaminase [EC:3.5.4.13] |
| K16033 | *asm12* | FADH2-dependent halogenase |
| K14599 | *dbfA1* | dibenzofuran dioxygenase subunit alpha [EC:1.14.12.-] |
| K14600 | *dbfA2* | dibenzofuran dioxygenase subunit beta [EC:1.14.12.-] |
| K15930 | *lndM2* | bifunctional oxygenase/reductase |
| K01937 | *pyrG, CTPS* | CTP synthase [EC:6.3.4.2] |
| K11991 | *tadA* | tRNA(adenine34) deaminase [EC:3.5.4.33] |
| K14630 | *actVA5* | two-component flavin-dependent monooxygenase [EC:1.14.14.-] |
| K15950 | *dnrF, rdmE* | aklavinone 12-hydroxylase [EC:1.14.13.180] |
| K03465 | *thyX, thy1* | thymidylate synthase (FAD) [EC:2.1.1.148] |
| K00560 | *thyA, TYMS* | thymidylate synthase [EC:2.1.1.45] |
| K11784 | *mqnC* | cyclic dehypoxanthinyl futalosine synthase [EC:1.21.98.1] |
| K16930 | *cdat8* | tRNA(cytosine8) deaminase [EC:3.5.4.35] |
| K01486 | *ade* | adenine deaminase [EC:3.5.4.2] |
| K00558 | *DNMT1, dcm* | DNA (cytosine-5)-methyltransferase 1 [EC:2.1.1.37] |
| K17398 | *DNMT3A* | DNA (cytosine-5)-methyltransferase 3A [EC:2.1.1.37] |
| K01696 | *trpB* | tryptophan synthase beta chain [EC:4.2.1.20] |
| K06001 | *trpB* | tryptophan synthase beta chain [EC:4.2.1.20] |
| K01695 | *trpA* | tryptophan synthase alpha chain [EC:4.2.1.20] |
| K03393 | *pacX* | phenylalanine 3-monooxygenase [EC:1.14.16.7] |
| K12714 | *clohal* | clorobiocin biosynthesis protein Clo-hal |
| K13604 | *bchU* | bacteriochlorophyllide d C-20 methyltransferase [EC:2.1.1.333] |
| K14586 | *nmoAB* | 2-naphthoate monooxygenase |
| K15963 | *mtmMII* | C-methyltransferase [EC:2.1.1.-] |
| K01667 | *tnaA* | tryptophanase [EC:4.1.99.1] |
| K00502 | *TPH1_2* | tryptophan 5-monooxygenase [EC:1.14.16.4] |
| K00480 | *E1.14.13.1* | salicylate hydroxylase [EC:1.14.13.1] |
| K14338 | *cypD_E, CYP102A, CYP505* | cytochrome P450 / NADPH-cytochrome P450 reductase [EC:1.14.14.1 1.6.2.4] |
| K00493 | *XANG* | xanthocillin biosynthesis cytochrome P450 monooxygenase [EC:1.14.-.-] |
| K11945 | *phdF* | extradiol dioxygenase [EC:1.13.11.-] |
| K03183 | *ubiE* | demethylmenaquinone methyltransferase / 2-methoxy-6-polyprenyl-1,4-benzoquinol methylase [EC:2.1.1.163 2.1.1.201] |
| K13540 | *cobIJ* | precorrin-2 C20-methyltransferase / precorrin-3B C17-methyltransferase [EC:2.1.1.130 2.1.1.131] |
| K03394 | *cobI-cbiL* | precorrin-2/cobalt-factor-2 C20-methyltransferase [EC:2.1.1.130 2.1.1.151] |
| K15945 | *snoaL2* | C-1 hydroxylase |
| K01757 | *STR1* | strictosidine synthase [EC:3.5.99.13] |
| K01485 | *codA* | cytosine/creatinine deaminase [EC:3.5.4.1 3.5.4.21] |
| K00529 | *hcaD* | 3-phenylpropionate/trans-cinnamate dioxygenase ferredoxin reductase component [EC:1.18.1.3] |
| K16319 | *andAc* | anthranilate 1,2-dioxygenase large subunit [EC:1.14.12.1] |
| K16320 | *andAd* | anthranilate 1,2-dioxygenase small subunit [EC:1.14.12.1] |
| K05710 | *hcaC* | 3-phenylpropionate/trans-cinnamate dioxygenase ferredoxin component |
| K05600 | *antB* | anthranilate 1,2-dioxygenase (deaminating, decarboxylating) small subunit [EC:1.14.12.1] |
| K11311 | *antC* | anthranilate 1,2-dioxygenase reductase component [EC:1.18.1.-] |
| K05599 | *antA* | anthranilate 1,2-dioxygenase (deaminating, decarboxylating) large subunit [EC:1.14.12.1] |
| K08686 | *cbdA* | 2-halobenzoate 1,2-dioxygenase large subunit [EC:1.14.12.13] |
| K15752 | *carAc* | carbazole 1,9a-dioxygenase ferredoxin component |
| K15753 | *carAd* | carbazole 1,9a-dioxygenase ferredoxin reductase component |
| K15751 | *carAa* | carbazole 1,9a-dioxygenase [EC:1.14.12.22] |
| K01694 | *TRP* | tryptophan synthase [EC:4.2.1.20] |
| K00457 | *HPD, hppD* | 4-hydroxyphenylpyruvate dioxygenase [EC:1.13.11.27] |
| K16249 | *dmpK, poxA, tomA0* | phenol/toluene 2-monooxygenase (NADH) P0/A0 |
| K16244 | *dmpM, poxC, tomA2* | phenol/toluene 2-monooxygenase (NADH) P2/A2 [EC:1.14.13.244 1.14.13.243] |
| K16245 | *dmpO, poxE, tomA4* | phenol/toluene 2-monooxygenase (NADH) P4/A4 [EC:1.14.13.244 1.14.13.243] |
| K16246 | *dmpP, poxF, tomA5* | phenol/toluene 2-monooxygenase (NADH) P5/A5 [EC:1.14.13.244 1.14.13.243] |
| K16242 | *dmpN, poxD, tomA3* | phenol/toluene 2-monooxygenase (NADH) P3/A3 [EC:1.14.13.244 1.14.13.243] |
| K16243 | *dmpL, poxB, tomA1* | phenol/toluene 2-monooxygenase (NADH) P1/A1 [EC:1.14.13.244 1.14.13.243] |
| K03380 | *E1.14.13.7* | phenol 2-monooxygenase (NADPH) [EC:1.14.13.7] |
| K15761 | *tmoB, tbuU, touB* | toluene monooxygenase system protein B [EC:1.14.13.236 1.14.13.-] |
| K15765 | *tmoF, tbuC, touF* | toluene monooxygenase electron transfer component [EC:1.18.1.3] |
| K15760 | *tmoA, tbuA1, touA* | toluene monooxygenase system protein A [EC:1.14.13.236 1.14.13.-] |
| K15762 | *tmoC, tbuB, touC* | toluene monooxygenase system ferredoxin subunit |
| K15763 | *tmoD, tbuV, touD* | toluene monooxygenase system protein D [EC:1.14.13.236 1.14.13.-] |
| K15764 | *tmoE, tbuA2, touE* | toluene monooxygenase system protein E [EC:1.14.13.236 1.14.13.-] |
| K16901 | *K16901* | anthranilate 3-monooxygenase (FAD) / 4-hydroxyphenylacetate 3-monooxygenase [EC:1.14.14.8 1.14.14.9] |
| K14333 | *DHBD* | 2,3-dihydroxybenzoate decarboxylase [EC:4.1.1.46] |
| K00486 | *KMO* | kynurenine 3-monooxygenase [EC:1.14.13.9] |
| K05712 | *mhpA* | 3-(3-hydroxy-phenyl)propionate hydroxylase [EC:1.14.13.127] |
| K01612 | *bsdC* | vanillate/4-hydroxybenzoate decarboxylase subunit C [EC:4.1.1.- 4.1.1.61] |
| K03688 | *ubiB, aarF* | ubiquinone biosynthesis protein |
| K00505 | *TYR* | tyrosinase [EC:1.14.18.1] |
| K15767 | *absAb* | 2-aminobenzenesulfonate 2,3-dioxygenase subunit beta [EC:1.14.12.14] |
| K15766 | *absAa* | 2-aminobenzenesulfonate 2,3-dioxygenase subunit alpha [EC:1.14.12.14] |
| K10676 | *tfdB* | 2,4-dichlorophenol 6-monooxygenase [EC:1.14.13.20] |
| K01668 | *E4.1.99.2* | tyrosine phenol-lyase [EC:4.1.99.2] |
| K04109 | *hcrB, hbaD* | 4-hydroxybenzoyl-CoA reductase subunit beta [EC:1.1.7.1] |
| K04107 | *hcrC, hbaB* | 4-hydroxybenzoyl-CoA reductase subunit gamma [EC:1.1.7.1] |
| K04108 | *hcrA, hbaC* | 4-hydroxybenzoyl-CoA reductase subunit alpha [EC:1.1.7.1] |
| K14583 | *nahC* | 1,2-dihydroxynaphthalene dioxygenase [EC:1.13.11.56] |
| K00500 | *phhA, PAH* | phenylalanine-4-hydroxylase [EC:1.14.16.1] |
| K07824 | *CYP53A1* | benzoate 4-monooxygenase [EC:1.14.14.92] |
| K15239 | *linD* | 2,5-dichlorohydroquinone reductive dechlorinase [EC:2.5.1.-] |
| K00484 | *hpaC* | flavin reductase (NADH) [EC:1.5.1.36] |
| K00483 | *hpaB* | 4-hydroxyphenylacetate 3-monooxygenase [EC:1.14.14.9] |
| K15241 | *pcpC* | tetrachlorohydroquinone reductive dehalogenase [EC:1.21.4.5] |
| K00422 | *E1.10.3.1* | polyphenol oxidase [EC:1.10.3.1] |
| K00481 | *pobA* | p-hydroxybenzoate 3-monooxygenase [EC:1.14.13.2] |
| K12706 | *novC* | novobiocin biosynthesis protein NovC |
| K12705 | *novO, couO* | 8-demethylnovobiocic acid C8-methyltransferase [EC:2.1.1.284] |
| K13387 | *CYP80A1* | berbamunine synthase [EC:1.14.19.66] |
| K03182 | *ubiD* | 4-hydroxy-3-polyprenylbenzoate decarboxylase [EC:4.1.1.98] |
| K03186 | *ubiX, bsdB, PAD1* | flavin prenyltransferase [EC:2.5.1.129] |
| K14974 | *nicC* | 6-hydroxynicotinate 3-monooxygenase [EC:1.14.13.114] |
| K04102 | *pht5* | 4,5-dihydroxyphthalate decarboxylase [EC:4.1.1.55] |
| K13385 | *CYP80B1* | N-methylcoclaurine 3'-monooxygenase [EC:1.14.14.102] |
| K13382 | *NCS* | (S)-norcoclaurine synthase [EC:3.5.99.14] |
| K15359 | *hspA* | 6-hydroxy-3-succinoylpyridine 3-monooxygenase [EC:1.14.13.163] |
| K15063 | *ligW* | 5-carboxyvanillate decarboxylase |
| K16048 | *hsaB* | 3-hydroxy-9,10-secoandrosta-1,3,5(10)-triene-9,17-dione monooxygenase reductase component [EC:1.5.1.-] |
| K16047 | *hsaA* | 3-hydroxy-9,10-secoandrosta-1,3,5(10)-triene-9,17-dione monooxygenase [EC:1.14.14.12] |
| K03391 | *pcpB* | pentachlorophenol monooxygenase [EC:1.14.13.50] |
| K12707 | *novQ, cloQ* | 4-hydroxyphenylpyruvate 3-dimethylallyltransferase [EC:2.5.1.111] |
| K14253 | *oxyE* | 6-methylpretetramide 4-monooxygenase [EC:1.14.13.232] |
| K14520 | *hapE* | 4-hydroxyacetophenone monooxygenase [EC:1.14.13.84] |
| K13565 | *LEPGT* | 4-hydroxybenzoate geranyltransferase [EC:2.5.1.93] |
| K12502 | *VTE3, APG1* | MPBQ/MSBQ methyltransferase [EC:2.1.1.295] |
| K06125 | *COQ2* | 4-hydroxybenzoate polyprenyltransferase [EC:2.5.1.39] |
| K03179 | *ubiA* | 4-hydroxybenzoate polyprenyltransferase [EC:2.5.1.39] |
| K10026 | *queE* | 7-carboxy-7-deazaguanine synthase [EC:4.3.99.3] |
| K09833 | *HPT, HGGT, ubiA* | homogentisate phytyltransferase / homogentisate geranylgeranyltransferase [EC:2.5.1.115 2.5.1.116] |
| K00767 | *nadC, QPRT* | nicotinate-nucleotide pyrophosphorylase (carboxylating) [EC:2.4.2.19] |
| K13421 | *UMPS* | uridine monophosphate synthetase [EC:2.4.2.10 4.1.1.23] |
| K01591 | *pyrF* | orotidine-5'-phosphate decarboxylase [EC:4.1.1.23] |
| K00480 | *E1.14.13.1* | salicylate hydroxylase [EC:1.14.13.1] |
| K15936 | *urdGT2* | glycosyltransferase [EC:2.4.1.-] |
| K13225 | *BX4, CYP71C1, CYP71C6* | 3-hydroxyindolin-2-one monooxygenase [EC:1.14.14.109] |
| K01587 | *PAICS* | phosphoribosylaminoimidazole carboxylase / phosphoribosylaminoimidazole-succinocarboxamide synthase [EC:4.1.1.21 6.3.2.6] |
| K11808 | *ADE2* | phosphoribosylaminoimidazole carboxylase [EC:4.1.1.21] |
| K12696 | *CYP71D12* | tabersonine 16-hydroxylase [EC:1.14.14.103] |
| K14333 | *DHBD* | 2,3-dihydroxybenzoate decarboxylase [EC:4.1.1.46] |
| K01612 | *bsdC* | vanillate/4-hydroxybenzoate decarboxylase subunit C [EC:4.1.1.- 4.1.1.61] |
| K13267 | *CYP71D9, F6H* | flavonoid 6-hydroxylase [EC:1.14.13.-] |
| K13389 | *CYP80G2* | (S)-corytuberine synthase [EC:1.14.19.51] |
| K16319 | *andAc* | anthranilate 1,2-dioxygenase large subunit [EC:1.14.12.1] |
| K05710 | *hcaC* | 3-phenylpropionate/trans-cinnamate dioxygenase ferredoxin component |
| K00529 | *hcaD* | 3-phenylpropionate/trans-cinnamate dioxygenase ferredoxin reductase component [EC:1.18.1.3] |
| K05600 | *antB* | anthranilate 1,2-dioxygenase (deaminating, decarboxylating) small subunit [EC:1.14.12.1] |
| K08686 | *cbdA* | 2-halobenzoate 1,2-dioxygenase large subunit [EC:1.14.12.13] |
| K11311 | *antC* | anthranilate 1,2-dioxygenase reductase component [EC:1.18.1.-] |
| K05599 | *antA* | anthranilate 1,2-dioxygenase (deaminating, decarboxylating) large subunit [EC:1.14.12.1] |
| K16320 | *andAd* | anthranilate 1,2-dioxygenase small subunit [EC:1.14.12.1] |
| K16330 | *K16330* | pseudouridylate synthase / pseudouridine kinase [EC:4.2.1.70 2.7.1.83] |
| K01718 | *pel* | pectate lyase [EC:4.2.2.2] |
| K16329 | *psuG* | pseudouridylate synthase [EC:4.2.1.70] |
| K13260 | *CYP81E* | isoflavone/4'-methoxyisoflavone 2'-hydroxylase [EC:1.14.14.90 1.14.14.89] |
| K13229 | *BX6* | 2,4-dihydroxy-1,4-benzoxazin-3-one-glucoside dioxygenase [EC:1.14.11.59] |
| K04102 | *pht5* | 4,5-dihydroxyphthalate decarboxylase [EC:4.1.1.55] |
| K13257 | *CYP93C* | 2-hydroxyisoflavanone synthase [EC:1.14.14.87] |
| K03186 | *ubiX, bsdB, PAD1* | flavin prenyltransferase [EC:2.5.1.129] |
| K03182 | *ubiD* | 4-hydroxy-3-polyprenylbenzoate decarboxylase [EC:4.1.1.98] |
| K05928 | *E2.1.1.95* | tocopherol O-methyltransferase [EC:2.1.1.95] |
| K14974 | *nicC* | 6-hydroxynicotinate 3-monooxygenase [EC:1.14.13.114] |
| K15063 | *ligW* | 5-carboxyvanillate decarboxylase |
| K16033 | *asm12* | FADH2-dependent halogenase |
| K15932 | *lndZ4, lanZ4* | reductase LndZ4/LanZ4 |
| K15933 | *lndZ5, lanZ5* | oxygenase LndZ5/LanZ5 |
| K14257 | *ctcP, cts4, prnC* | tetracycline 7-halogenase / FADH2 O2-dependent halogenase [EC:1.14.19.49 1.14.19.-] |
| K15930 | *lndM2* | bifunctional oxygenase/reductase |
| K03465 | *thyX, thy1* | thymidylate synthase (FAD) [EC:2.1.1.148] |
| K13998 | *DHFR-TS* | dihydrofolate reductase / thymidylate synthase [EC:1.5.1.3 2.1.1.45] |
| K00560 | *thyA, TYMS* | thymidylate synthase [EC:2.1.1.45] |
| K15950 | *dnrF, rdmE* | aklavinone 12-hydroxylase [EC:1.14.13.180] |
| K14630 | *actVA5* | two-component flavin-dependent monooxygenase [EC:1.14.14.-] |
| K15506 | *CYP98A8* | cytochrome P450 family 98 subfamily A8 [EC:1.14.13.-] |
| K13498 | *trpCF* | indole-3-glycerol phosphate synthase / phosphoribosylanthranilate isomerase [EC:4.1.1.48 5.3.1.24] |
| K13501 | *TRP1* | anthranilate synthase / indole-3-glycerol phosphate synthase / phosphoribosylanthranilate isomerase [EC:4.1.3.27 4.1.1.48 5.3.1.24] |
| K01656 | *TRP3* | anthranilate synthase / indole-3-glycerol phosphate synthase [EC:4.1.3.27 4.1.1.48] |
| K01609 | *trpC* | indole-3-glycerol phosphate synthase [EC:4.1.1.48] |
| K11784 | *mqnC* | cyclic dehypoxanthinyl futalosine synthase [EC:1.21.98.1] |
| K07412 | *CYP2B* | cytochrome P450 family 2 subfamily B [EC:1.14.14.1] |
| K07409 | *CYP1A2* | cytochrome P450 family 1 subfamily A2 [EC:1.14.14.1] |
| K00558 | *DNMT1, dcm* | DNA (cytosine-5)-methyltransferase 1 [EC:2.1.1.37] |
| K17399 | *DNMT3B* | DNA (cytosine-5)-methyltransferase 3B [EC:2.1.1.37] |
| K17398 | *DNMT3A* | DNA (cytosine-5)-methyltransferase 3A [EC:2.1.1.37] |
| K01695 | *trpA* | tryptophan synthase alpha chain [EC:4.2.1.20] |
| K06001 | *trpB* | tryptophan synthase beta chain [EC:4.2.1.20] |
| K01696 | *trpB* | tryptophan synthase beta chain [EC:4.2.1.20] |
| K07410 | *CYP1B1* | cytochrome P450 family 1 subfamily B1 [EC:1.14.14.1] |
| K07408 | *CYP1A1* | cytochrome P450 family 1 subfamily A1 [EC:1.14.14.1] |
| K05280 | *CYP75B1* | flavonoid 3'-monooxygenase [EC:1.14.14.82] |
| K13083 | *CYP75A* | flavonoid 3',5'-hydroxylase [EC:1.14.14.81] |
| K03393 | *pacX* | phenylalanine 3-monooxygenase [EC:1.14.16.7] |
| K12714 | *clohal* | clorobiocin biosynthesis protein Clo-hal |
| K00157 | *AOX* | aldehyde oxidase [EC:1.2.3.1] |
| K14586 | *nmoAB* | 2-naphthoate monooxygenase |
| K13604 | *bchU* | bacteriochlorophyllide d C-20 methyltransferase [EC:2.1.1.333] |
| K00502 | *TPH1_2* | tryptophan 5-monooxygenase [EC:1.14.16.4] |
| K01667 | *tnaA* | tryptophanase [EC:4.1.99.1] |
| K15963 | *mtmMII* | C-methyltransferase [EC:2.1.1.-] |
| K00493 | *XANG* | xanthocillin biosynthesis cytochrome P450 monooxygenase [EC:1.14.-.-] |
| K14338 | *cypD_E, CYP102A, CYP505* | cytochrome P450 / NADPH-cytochrome P450 reductase [EC:1.14.14.1 1.6.2.4] |
| K03183 | *ubiE* | demethylmenaquinone methyltransferase / 2-methoxy-6-polyprenyl-1,4-benzoquinol methylase [EC:2.1.1.163 2.1.1.201] |
| K13222 | *BX1, IGL* | indole-3-glycerol-phosphate lyase [EC:4.1.2.8] |
| K07424 | *CYP3A* | cytochrome P450 family 3 subfamily A [EC:1.14.14.1] |
| K15945 | *snoaL2* | C-1 hydroxylase |
| K03394 | *cobI-cbiL* | precorrin-2/cobalt-factor-2 C20-methyltransferase [EC:2.1.1.130 2.1.1.151] |
| K13540 | *cobIJ* | precorrin-2 C20-methyltransferase / precorrin-3B C17-methyltransferase [EC:2.1.1.130 2.1.1.131] |
| K01757 | *STR1* | strictosidine synthase [EC:3.5.99.13] |
| K00457 | *HPD, hppD* | 4-hydroxyphenylpyruvate dioxygenase [EC:1.13.11.27] |
| K16246 | *dmpP, poxF, tomA5* | phenol/toluene 2-monooxygenase (NADH) P5/A5 [EC:1.14.13.244 1.14.13.243] |
| K16243 | *dmpL, poxB, tomA1* | phenol/toluene 2-monooxygenase (NADH) P1/A1 [EC:1.14.13.244 1.14.13.243] |
| K16249 | *dmpK, poxA, tomA0* | phenol/toluene 2-monooxygenase (NADH) P0/A0 |
| K16242 | *dmpN, poxD, tomA3* | phenol/toluene 2-monooxygenase (NADH) P3/A3 [EC:1.14.13.244 1.14.13.243] |
| K16244 | *dmpM, poxC, tomA2* | phenol/toluene 2-monooxygenase (NADH) P2/A2 [EC:1.14.13.244 1.14.13.243] |
| K16245 | *dmpO, poxE, tomA4* | phenol/toluene 2-monooxygenase (NADH) P4/A4 [EC:1.14.13.244 1.14.13.243] |
| K03380 | *E1.14.13.7* | phenol 2-monooxygenase (NADPH) [EC:1.14.13.7] |
| K16901 | *K16901* | anthranilate 3-monooxygenase (FAD) / 4-hydroxyphenylacetate 3-monooxygenase [EC:1.14.14.8 1.14.14.9] |
| K15760 | *tmoA, tbuA1, touA* | toluene monooxygenase system protein A [EC:1.14.13.236 1.14.13.-] |
| K15765 | *tmoF, tbuC, touF* | toluene monooxygenase electron transfer component [EC:1.18.1.3] |
| K15762 | *tmoC, tbuB, touC* | toluene monooxygenase system ferredoxin subunit |
| K15761 | *tmoB, tbuU, touB* | toluene monooxygenase system protein B [EC:1.14.13.236 1.14.13.-] |
| K15764 | *tmoE, tbuA2, touE* | toluene monooxygenase system protein E [EC:1.14.13.236 1.14.13.-] |
| K15763 | *tmoD, tbuV, touD* | toluene monooxygenase system protein D [EC:1.14.13.236 1.14.13.-] |
| K10437 | *PHAA, PHACA, CYP504A1* | phenylacetate 2-hydroxylase [EC:1.14.14.54] |
| K05712 | *mhpA* | 3-(3-hydroxy-phenyl)propionate hydroxylase [EC:1.14.13.127] |
| K00487 | *CYP73A* | trans-cinnamate 4-monooxygenase [EC:1.14.14.91] |
| K00486 | *KMO* | kynurenine 3-monooxygenase [EC:1.14.13.9] |
| K15753 | *carAd* | carbazole 1,9a-dioxygenase ferredoxin reductase component |
| K15751 | *carAa* | carbazole 1,9a-dioxygenase [EC:1.14.12.22] |
| K14599 | *dbfA1* | dibenzofuran dioxygenase subunit alpha [EC:1.14.12.-] |
| K15752 | *carAc* | carbazole 1,9a-dioxygenase ferredoxin component |
| K14600 | *dbfA2* | dibenzofuran dioxygenase subunit beta [EC:1.14.12.-] |
| K00505 | *TYR* | tyrosinase [EC:1.14.18.1] |
| K03688 | *ubiB, aarF* | ubiquinone biosynthesis protein |
| K10676 | *tfdB* | 2,4-dichlorophenol 6-monooxygenase [EC:1.14.13.20] |
| K01694 | *TRP* | tryptophan synthase [EC:4.2.1.20] |
| K04109 | *hcrB, hbaD* | 4-hydroxybenzoyl-CoA reductase subunit beta [EC:1.1.7.1] |
| K04108 | *hcrA, hbaC* | 4-hydroxybenzoyl-CoA reductase subunit alpha [EC:1.1.7.1] |
| K04107 | *hcrC, hbaB* | 4-hydroxybenzoyl-CoA reductase subunit gamma [EC:1.1.7.1] |
| K03782 | *katG* | catalase-peroxidase [EC:1.11.1.21] |
| K03781 | *katE, CAT, catB, srpA* | catalase [EC:1.11.1.6] |
| K10438 | *PHACB, CYP504B1* | 3-hydroxyphenylacetate 6-hydroxylase [EC:1.14.13.63] |
| K15239 | *linD* | 2,5-dichlorohydroquinone reductive dechlorinase [EC:2.5.1.-] |
| K09755 | *CYP84A, F5H* | ferulate-5-hydroxylase [EC:1.14.-.-] |
| K01668 | *E4.1.99.2* | tyrosine phenol-lyase [EC:4.1.99.2] |
| K00500 | *phhA, PAH* | phenylalanine-4-hydroxylase [EC:1.14.16.1] |
| K07824 | *CYP53A1* | benzoate 4-monooxygenase [EC:1.14.14.92] |
| K00483 | *hpaB* | 4-hydroxyphenylacetate 3-monooxygenase [EC:1.14.14.9] |
| K00484 | *hpaC* | flavin reductase (NADH) [EC:1.5.1.36] |
| K15241 | *pcpC* | tetrachlorohydroquinone reductive dehalogenase [EC:1.21.4.5] |
| K00422 | *E1.10.3.1* | polyphenol oxidase [EC:1.10.3.1] |
| K00501 | *TH* | tyrosine 3-monooxygenase [EC:1.14.16.2] |
| K15767 | *absAb* | 2-aminobenzenesulfonate 2,3-dioxygenase subunit beta [EC:1.14.12.14] |
| K15766 | *absAa* | 2-aminobenzenesulfonate 2,3-dioxygenase subunit alpha [EC:1.14.12.14] |
| K00431 | *TPO* | thyroid peroxidase [EC:1.11.1.8] |
| K00481 | *pobA* | p-hydroxybenzoate 3-monooxygenase [EC:1.14.13.2] |
| K09754 | *CYP98A, C3'H* | 5-O-(4-coumaroyl)-D-quinate 3'-monooxygenase [EC:1.14.14.96] |
| K12706 | *novC* | novobiocin biosynthesis protein NovC |
| K12705 | *novO, couO* | 8-demethylnovobiocic acid C8-methyltransferase [EC:2.1.1.284] |
| K13385 | *CYP80B1* | N-methylcoclaurine 3'-monooxygenase [EC:1.14.14.102] |
| K15359 | *hspA* | 6-hydroxy-3-succinoylpyridine 3-monooxygenase [EC:1.14.13.163] |
| K07415 | *CYP2E1* | cytochrome P450 family 2 subfamily E1 [EC:1.14.14.-] |
| K16047 | *hsaA* | 3-hydroxy-9,10-secoandrosta-1,3,5(10)-triene-9,17-dione monooxygenase [EC:1.14.14.12] |
| K16048 | *hsaB* | 3-hydroxy-9,10-secoandrosta-1,3,5(10)-triene-9,17-dione monooxygenase reductase component [EC:1.5.1.-] |
| K03391 | *pcpB* | pentachlorophenol monooxygenase [EC:1.14.13.50] |
| K13382 | *NCS* | (S)-norcoclaurine synthase [EC:3.5.99.14] |
| K12707 | *novQ, cloQ* | 4-hydroxyphenylpyruvate 3-dimethylallyltransferase [EC:2.5.1.111] |
| K14520 | *hapE* | 4-hydroxyacetophenone monooxygenase [EC:1.14.13.84] |
| K13565 | *LEPGT* | 4-hydroxybenzoate geranyltransferase [EC:2.5.1.93] |
| K07414 | *CYP2D* | cytochrome P450 family 2 subfamily D [EC:1.14.14.1] |
| K12502 | *VTE3, APG1* | MPBQ/MSBQ methyltransferase [EC:2.1.1.295] |
| K09833 | *HPT, HGGT, ubiA* | homogentisate phytyltransferase / homogentisate geranylgeranyltransferase [EC:2.5.1.115 2.5.1.116] |
| K03179 | *ubiA* | 4-hydroxybenzoate polyprenyltransferase [EC:2.5.1.39] |
| K06125 | *COQ2* | 4-hydroxybenzoate polyprenyltransferase [EC:2.5.1.39] |
| K01721 | *nthA* | nitrile hydratase subunit alpha [EC:4.2.1.84] |
| K06920 | *queC* | 7-cyano-7-deazaguanine synthase [EC:6.3.4.20] |
| K01501 |  | nitrilase [EC:3.5.5.1] |

**Table S5.** Enzymes computed by E-zyme2 whose coding genes were mined from the FLU metagenomes

| **Enzyme** | **KEGG Orthology** | **EC Number** |
| --- | --- | --- |
| **Sub-product: CID139595326** | | |
| 3-phenylpropionate/trans-cinnamate dioxygenase | K00529  K05710 | 1.18.1.3 |
| Dihydroorotate dehydrogenase | K00226  K00254 | 1.3.98.1  1.3.5.2 |
| Urocanate hydratase | K01712  K02609  K02610  K02611  K02612  K02613 | 4.2.1.49 |
| Anthranilate synthase | K01657  K01658  K13503 | 4.1.3.27 |
| Anthranilate phosphoribosyltransferase | K13497 | 2.4.2.18 |
| Dihydrofolate reductase | K00287 | 1.5.1.3 |
| *para*-aminobenzoate synthetase | K03342 | 2.6.1.85 |
| 4-amino-4-deoxychorismate lyase | K02619 | 4.1.3.38 |
| Chorismate lyase | K03181 | 4.1.3.40 |
| 2-octaprenyl-6-methoxyphenol hydroxylase | K03185 | 1.14.13.- |
| *O*-succinylbenzoate synthase | K02549 | 4.2.1.113 |
| Isochorismate pyruvate | K04782 | 4.2.99.21 |
| Benzoate/toluate 1,2-dioxygenase | K05549  K05550  K05784 | 1.14.12.10  1.18.1.- |
| Benzoyl-CoA reductase | K04113 | 1.3.7.8 |
| Anthraniloyl-CoA monooxygenase | K09461 | 1.14.13.40 |
| Naphthalene 1,2-dioxygenase | K14578  K14581 | 1.18.1.7 |
| Ethylbenzene dioxygenase | K14748  K14749 | 1.14.12.- |
| **Sub-product: CID139594537** | | |
| Catalase | K03781 | 1.11.1.6 |
| 3-phenylpropionate/trans-cinnamate dioxygenase | K00529  K05710 | 1.18.1.3  1.6.2.4 |
| NADPH-cytochrome P450 reductase | K14338 | 1.14.14.1 |
| Hypoxanthine phosphoribosyltransferase | K00760 | 2.4.2.8 |
| IMP dehydrogenase | K00088 | 1.1.1.205 |
| Adenylosuccinate synthase | K01939 | 6.3.4.4 |
| Adenosine deaminase | K01488 | 3.5.4.4 |
| Adenine deaminase | K01486 | 3.5.4.2 |
| CTP synthase | K01937 | 6.3.4.2 |
| Pseudouridylate synthase | K16329 | 4.2.1.70 |
| Cytidine deaminase | K01489 | 3.5.4.5 |
| Cytosine/creatinine deaminase | K01485 | 3.5.4.1  3.5.4.21 |
| dCTP deaminase | K01494 | 3.5.4.13 |
| dCMP deaminase | K01493 | 3.5.4.12 |
| Thymidylate synthase | K00560  K03465 | 2.1.1.45  2.1.1.148 |
| Tryptophan synthase | K01695  K01696  K06001 | 4.2.1.20 |
| DNA (cytosine-5)-methyltransferase | K00558 | 2.1.1.37 |
| 4-hydroxyphenylpyruvate dioxygenase | K00457 | 1.13.11.27 |
| 4-hydroxyphenylacetate 3-monooxygenase | K00483 | 1.14.14.9 |
| Flavin reductase | K00484 | 1.5.1.36 |
| Tyrosine phenol-lyase | K01668 | 4.1.99.2 |
| Phenylalanine-4-hydroxylase | K00500 | 1.14.16.1 |
| Catalase-peroxidase | K03782 | 1.11.1.21 |
| 3-(3-hydroxyphenyl)propionate hydroxylase | K05712 | 1.14.13.127 |
| Kynurenine 3-monooxygenase | K00486 | 1.14.13.9 |
| Tryptophanase | K01667 | 4.1.99.1 |
| Flavin prenyltransferase | K03186 | 2.5.1.129 |
| Nicotinate-nucleotide pyrophosphorylase | K00767 | 2.4.2.19 |
| 6-hydroxynicotinate 3-monooxygenase | K14974 | 1.14.13.114 |
| 6-hydroxy-3-succinoylpyridine 3-monooxygenase | K15359 | 1.14.13.163 |
| 7-carboxy-7-deazaguanine synthase | K10026 | 4.3.99.3 |
| Precorrin-2/cobalt-factor-2 C20-methyltransferase | K03394 | 2.1.1.130  2.1.1.151 |
| 4-hydroxybenzoate polyprenyltransferase | K03179 | 2.5.1.39 |
| 4-hydroxy-3-polyprenylbenzoate decarboxylase | K03182 | 4.1.1.98 |
| Demethylmenaquinone methyltransferase  2-methoxy-6-polyprenyl-1,4-benzoquinol methylase | K03183 | 2.1.1.163  2.1.1.201 |
| Cyclic dehypoxanthinyl futalosine synthase | K11784 | 1.21.98.1 |
| Phenol/toluene 2-monooxygenase | K16249  K16243  K16244  K16242  K16245  K16246 | 1.14.13.243 |
| 2,3-dihydroxybenzoate decarboxylase | K14333 | 4.1.1.46 |
| *p*-hydroxybenzoate 3-monooxygenase | K00481 | 1.14.13.2 |
| Vanillate/4-hydroxybenzoate | K01612 | 4.1.1.61 |
| Anthranilate 1,2-dioxygenase | K05599  K05600  K11311  K16319  K16320 | 1.14.12.1 |
| 5-carboxyvanillate decarboxylase | K15063 |  |
| 2,4-dichlorophenol 6-monooxygenase | K10676 | 1.14.13.20 |
| Toluene monooxygenase | K15763  K15764 | 1.14.13.236 |
| 4-hydroxyacetophenone monooxygenase | K14520 | 1.14.13.84 |
| 4,5-dihydroxyphthalate decarboxylase | K04102 | 4.1.1.55 |
| 3-hydroxy-9,10-secoandrosta-1,3,5(10)-triene-9,17-dione monooxygenase | K16047  K16048 | 1.14.14.12 |
| tRNA deaminase | K11991 | 3.5.4.33 |
| **Sub-product: CID2774067** | | |
| Catalase | K03781 | 1.11.1.6 |
| 3-phenylpropionate/trans-cinnamate dioxygenase | K00529  K05710 | 1.18.1.3 |
| NADPH-cytochrome P450 | K14338 | 1.14.14.1  1.6.2.4 |
| Orotidine-5'-phosphate decarboxylase | K01591 | 4.1.1.23 |
| Pseudouridylate synthase | K16329 | 4.2.1.70 |
| Thymidylate synthase | K00560  K03465 | 2.1.1.45  2.1.1.148 |
| Tryptophan synthase | K01695  K01696  K06001 | 4.2.1.20 |
| DNA (cytosine-5)-methyltransferase | K00558 | 2.1.1.37 |
| 4-hydroxyphenylpyruvate dioxygenase | K00457 | 1.13.11.27 |
| 4-hydroxyphenylacetate 3-monooxygenase | K00483 | 1.14.14.9 |
| Flavin reductase | K00484 | 1.5.1.36 |
| Tyrosine phenol-lyase | K01668 | 4.1.99.2 |
| Phenylalanine-4-hydroxylase | K00500 | 1.14.16.1 |
| Catalase-peroxidase | K03782 | 1.11.1.21 |
| 3-(3-hydroxy-phenyl)propionate hydroxylase | K05712 | 1.14.13.127 |
| Kynurenine 3-monooxygenase | K00486 | 1.14.13.9 |
| Tryptophanase | K01667 | 4.1.99.1 |
| Indole-3-glycerol phosphate synthase | K01609 | 4.1.1.48 |
| Flavin prenyltransferase | K03186 | 2.5.1.129 |
| Nicotinate-nucleotide pyrophosphorylase | K00767 | 2.4.2.19 |
| 6-hydroxynicotinate 3-monooxygenase | K14974 | 1.14.13.114 |
| 6-hydroxy-3-succinoylpyridine 3-monooxygenase | K15359 | 1.14.13.163 |
| Precorrin-2/cobalt-factor-2 C20-methyltransferase | K03394 | 2.1.1.130  2.1.1.151 |
| 4-hydroxybenzoate polyprenyltransferase | K03179 | 2.5.1.39 |
| 4-hydroxy-3-polyprenylbenzoate decarboxylase | K03182 | 4.1.1.98 |
| Demethylmenaquinone methyltransferase  2-methoxy-6-polyprenyl-1,4-benzoquinol methylase | K03183 | 2.1.1.163  2.1.1.201 |
| Cyclic dehypoxanthinyl futalosine synthase | K11784 | 1.21.98.1 |
| Phenol/toluene 2-monooxygenase | K16249  K16243  K16244  K16242  K16245  K16246 | 1.14.13.243 |
| 2,3-dihydroxybenzoate decarboxylase | K14333 | 4.1.1.46 |
| *p*-hydroxybenzoate 3-monooxygenase | K00481 | 1.14.13.2 |
| Phenol 2-monooxygenase | K03380 | 1.14.13.7 |
| Vanillate/4-hydroxybenzoate decarboxylase | K01612 | 4.1.1.61 |
| Anthranilate 1,2-dioxygenase | K05599  K05600  K11311  K16319  K16320 | 1.14.12.1 |
| 5-carboxyvanillate decarboxylase | K15063 |  |
| 2,5-dichlorohydroquinone reductive dechlorinase | K15239 | 2.5.1.- |
| 2,4-dichlorophenol 6-monooxygenase | K10676 | 1.14.13.20 |
| Toluene monooxygenase | K15763  K15764 | 1.14.13.236 |
| 4-hydroxyacetophenone monooxygenase | K14520 | 1.14.13.84 |
| 4,5-dihydroxyphthalate decarboxylase | K04102 | 4.1.1.55 |
| 3-hydroxy-9,10-secoandrosta-1,3,5(10)-triene-9,17-dione monooxygenase | K16047  K16048 | 1.14.14.12 |
| **Sub-product: CID90135849** | | |
| Nitrile hydratase | K01721 | 4.2.1.84 |
| **Sub-product: CID153925794** | | |
| Nitrilase | K01501 | 3.5.5.1 |
| 7-cyano-7-deazaguanine synthase | K06920 | 6.3.4.20 |

**Table S6.** Enzymes computed by E-zyme2 whose coding genes were mined from the EPO metagenomes

| **Enzyme** | **KEGG Orthology** | **Enzyme Classification** |
| --- | --- | --- |
| **Sub-product: CID13602778** | | |
| Soluble epoxide hydrolase | K08726 | 3.3.2.10 |
| **Sub-product: CID139597444** | | |
| Naphtalene 1,2-dioxygenase | K1458  K14578 | 1.18.1.7 |
| Dibenzofuran dioxygenase | K14599  K14600 | 1.14.12.- |
| 2-hydroxyisoflavone reductase | K05281 | 1.3.1.45 |
| Microsomal epoxide hydrolase | K01253 | 3.3.2.9 |
| Tyrosinase | K00505 | 1.14.18.1 |
| PAH dioxygenase | K11944  K11943 | 1.13.11.- |
| 4-hydroxymandelate synthase | K16421 | 1.13.11.46 |
| Magnesium-protoporphyrin IX monomethyl ester (oxidative) cyclase | K04035 | 1.14.13.81 |
| Ethylbenzene hydroxylase | K17048  K17049  K10700 | 1.17.99.2 |
| Biphenyl 2,3-dioxygenase | K08689  K15750 | 1.14.12.18 |
| 3-phenylpropionate dioxygenase | K00529 | 1.18.1.3 |
| Styrene monooxygenase | K14482  K14481 | 1.5.1.- |
| 2-enoate reductase | K10797 | 1.3.1.31 |
| Phosphoribosylanthranilate isomerase | K01817 | 5.3.1.24 |
| **Sub-product: CID10870725** | | |
| 2-hydroxychromene-2-carboxylate isomerase | K14584 | 5.99.1.4 |
| **Sub-product: CID5216290** | | |
| 2-ketocyclohexanecarboxyl-CoA hydrolase | K07536 | 3.1.2.- |
| 6-oxocyclohex-1-ene-carbonyl-CoA hydrolase | K07539 | 3.7.1.21 |
| 3D-(3,5/4)-trihydroxycyclohexane-1,2-dione acylhydrolase | K03336 | 3.7.1.22 |
| **Sub-product: CID135436542** | | |
| Glutamate-1-semialdehyde 2,1-aminomutase | K01845 | 5.4.3.8 |
| Xanthine dehydrogenase | K00106  K13480  K11178  K13479  K00087  K11177  K13483  K13482  K13481 | 1.17.1.4 |
| Histamine N-methyltransferase | K00546 | 2.1.1.8 |
| Adenine deaminase | K01486 | 3.5.4.2 |
| IMP dehydrogenase | K00088 | 1.1.1.205 |
| Cytidine deaminase | K01489 | 3.5.4.5 |
| Guanine deaminase | K01487 | 3.5.4.3 |
| dCMP deaminase | K01493 | 3.5.4.12 |
| Diaminohydroxyphosphoribosylaminopyrimidine deaminase | K01498  K11752 | 3.5.4.26 |
| Adenosine deaminase | K01488 | 3.5.4.4 |
| GMP synthase | K01951 | 6.3.5.2 |
| dCTP deaminase | K01494 | 3.5.4.13 |
| CTP synthase | K01937 | 6.3.4.2 |
| Futalosine hydrolase | K11783 | 3.2.2.26 |
| Adenylosuccinate synthase | K01939 | 6.3.4.4 |
| dCTP deaminase (dump-forming) | K09887 | 3.5.4.30 |
| 7,8-didemethyl-8-hydroxy-5-deazariboflavin synthase | K11780 | 4.3.1.32 |
| 5-amino-6-(D-ribitylamino)uracil---L-tyrosine 4-hydroxyphenyl transferase | K11781 | 2.5.1.147 |
| tRNA deaminase | K11991  K16930 | 3.5.4.33  3.5.4.35 |
| Hypoxanthine phosphoribosyltransferase | K00760 | 2.4.2.8 |
| Purine-nucleoside phosphorylase | K03784  K03783 | 2.4.2.1 |
| Adenine phosphoribosyltransferase | K00759 | 2.4.2.7 |
| Ribosylpyrimidine nucleosidase | K10213 | 3.2.2.8 |
| Pyrimidine-nucleoside phosphorylase | K00756 | 2.4.2.2 |
| Thymidine phosphorylase | K00758 | 2.4.2.4 |
| Adenosylhomocysteine nucleosidase | K01243 | 3.2.2.9 |
| 5'-methylthioadenosine phosphorylase | K00772 | 2.4.2.28 |
| 5'-methylthioadenosine nucleosidase | K01244 | 3.2.2.16 |
| Purine nucleosidase | K01239 | 3.2.2.1 |
| Nicotinate-nucleotide pyrophosphorylase | K00767 | 2.4.2.19 |
| Nucleoside deoxyribosyltransferase | K08728 | 2.4.2.6 |
| AMP nucleosidase | K01241 | 3.2.2.4 |
| Uridine phosphorylase | K00757 | 2.4.2.3 |
| Xanthine phosphoribosyltransferase | K00769  K03816 | 2.4.2.22 |
| Alpha-D-ribose-1-methylphosphonate-5-triphosphate synthase | K06164  K06165  K06166  K05780 | 2.7.8.37 |
| Triphosphoribosyl-dephospho-coA synthase | K05966  K13927  K13930 | 2.4.2.52 |
| Nicotinamide phosphoribosyltransferase | K03462 | 2.4.2.12 |
| Nicotinate-nucleotide--dimethylbenzimidazole phosphoribosyltransferase | K00768 | 2.4.2.21 |
| tRNA-lysidine synthase | K04075 | 6.3.4.19 |
| Uridine monophosphate synthetase | K13421 | 2.4.2.10  4.1.11.23 |
| Uracil phosphoribosyltransferase | K00761 | 2.4.2.9 |
| Nicotinate phosphoribosyltransferase | K00763 | 6.3.4.21 |
| Orotate phosphoribosyltransferase | K00762 | 2.4.2.10 |
| 6,7-dimethyl-8-ribityllumazine synthase | K00794 | 2.5.1.78 |
| Riboflavin synthase | K00793 | 2.5.1.9 |
| Queuine tRNA-ribosyltransferase | K00773 | 2.4.2.29 |
| **Sub-product: CID5216290** | | |
| 2-ketocyclohexanecarboxyl-CoA hydrolase | K07536 | 3.1.2.- |
| 6-oxocyclohex-1-ene-carbonyl-CoA hydrolase | K07539 | 3.7.1.21 |
| 3D-(3,5/4)-trihydroxycyclohexane-1,2-dione acylhydrolase (decyclizing) | K03336 | 3.7.1.22 |
| 3-dehydroquinate synthase | K11646 | 1.4.1.24 |
